# Hidden deficiency under bright skies: Vitamin D prevalence and genetic associations in African Type 2 diabetes: A systematic review and meta-analysis

**DOI:** 10.1371/journal.pone.0354518

**Published:** 2026-07-24

**Authors:** Eyob Girma Abera, Samuel Alemu Himbaro, Surafel Worku Megersa, Tesfaye Adugna Leta, Ermias Habte Gebremichael

**Affiliations:** 1 Department of Public Health, Jimma University, Jimma, Oromia, Ethiopia; 2 Jimma University Clinical Trial Unit, Jimma, Oromia, Ethiopia; 3 Independent Medical Researcher, Addis Ababa, Ethiopia; 4 Department of Psychiatry, Saint Paul’s Hospital Millennium Medical College, Addis Ababa, Ethiopia; 5 Department of Biomedical Science, Jimma University, Jimma, Oromia, Ethiopia; 6 Department of Internal Medicine, Jimma University, Jimma, Oromia, Ethiopia; University of Connecticut, UNITED STATES OF AMERICA

## Abstract

**Background:**

Type 2 diabetes mellitus (T2DM) has become a significant health challenge in Africa, with rapidly increasing prevalence. Despite abundant sunlight, vitamin D deficiency (VDD) is prevalent and may influence glucose metabolism and insulin resistance. Genetic factors, including vitamin D receptor (VDR) polymorphisms, may modify these effects. This systematic review and meta-analysis aimed to estimate the prevalence of VDD and evaluate the association of VDR gene variants with T2DM among African populations.

**Methods:**

We systematically searched PubMed, Cochrane Library, ScienceDirect, Embase, Web of Science, and Google Scholar for observational studies published up to June 11, 2025, reporting VDD prevalence and VDR genetic associations in African individuals with T2DM. Data extraction and quality assessment followed Joanna Briggs Institute (JBI) and PRISMA 2020 guidelines. Pooled prevalence, mean difference and odds ratios (ORs) were calculated using random-effects meta-analysis. Heterogeneity, publication bias, sensitivity analyses, and meta-regression were performed. All statistical analyses were conducted using R version 4.3.1 (2023-06-16 ucrt).

**Results:**

Twenty-two eligible studies encompassing 3,447 participants from 11 African countries were included. The pooled prevalence of VDD among individuals with T2DM was 51% (95% CI: 36–65%), with substantial heterogeneity (I^2^ = 95.7%). Subgroup analyses showed highest prevalence in East Africa (56%). Vitamin D-deficient individuals had significantly higher HbA1c compared to sufficient individuals (mean difference = 0.89%; 95% CI: 0.06–1.72 p = 0.044; I^2^ = 0%, p = 0.49). The pooled analysis of VDR FokI polymorphisms did not demonstrate a significant association with T2DM risk (OR = 1.51; 95% CI: 0.23–9.73). No evidence of publication bias was detected.

**Conclusion:**

Vitamin D deficiency is highly prevalent in African populations with T2DM and is associated with poorer glycemic control. Current evidence is insufficient to conclude on VDR FokI polymorphisms and T2DM risk. These findings underscore the need for interventional trials to determine whether improving vitamin D status enhances glycemic outcomes; routine screening or supplementation recommendations remain premature.

**Registration number:**

CRD420251134616.

## Introduction

Type 2 diabetes mellitus (T2DM) has emerged as a significant public health concern in Africa, with its prevalence increasing rapidly across the continent. The International Diabetes Federation (IDF) reported that in 2021, approximately 24 million adults aged 20–79 years were living with diabetes in the African region, representing a prevalence of 4.5%. Alarmingly, more than half of these individuals remain undiagnosed, the highest proportion globally [1]. Projections indicate that the number of adults with diabetes in Africa will rise to 60 million by 2050, marking a 142% increase [2].

Recent evidence suggests a novel role of vitamin D in glucose homeostasis and insulin metabolism. Vitamin D modulates the immune system, regulates inflammatory cytokines, improves insulin secretion, and reduces insulin resistance. These mechanisms may influence the development and progression of T2DM [3]. In humans, the majority of vitamin D is synthesized in the skin upon exposure to ultraviolet B (UVB) radiation, while a smaller portion comes from dietary sources such as fatty fish, eggs, and fortified foods [4]. Serum 25-hydroxyvitamin D [25(OH)D] is the most reliable biomarker for assessing vitamin D status.

Concurrently, despite the continent’s abundant sunlight, vitamin D deficiency (VDD) has been identified as a prevalent condition in African populations, with studies reporting varying prevalence rates. For instance, a study in South Africa found a 28.5% prevalence of VDD among healthcare workers [5]. Various studies in Africa reported a heterogynous prevalence of VDD among individuals with T2DM, ranging from 3.3% [6] to 92.4% [7]. These discrepancies highlight the need for region-specific assessments to understand the extent of VDD and its potential implications for T2DM.

Genetic factors play a crucial role in the pathogenesis of T2DM. Research has identified several genetic polymorphisms associated with T2DM in African populations. In particular, variants in genes such as TCF7L2, TNF-α, and ENPP1 have been linked to increased susceptibility to T2DM [8]. Particularly the FokI polymorphism, may modify the effects of vitamin D on insulin secretion and sensitivity, potentially influencing glycemic control and insulin resistance in T2DM patients [9]. Evidence on the combined effects of vitamin D status and vitamin D receptor (VDR) genetic determinants in African populations is limited and heterogeneous, with inconsistent findings.

Understanding the combined effects of VDD and genetic predispositions on T2DM is essential for developing targeted prevention and treatment strategies. Therefore, this systematic review aims to synthesize existing literature on the prevalence of VDD and its genetic associations among African populations with T2DM. By doing so, we seek to provide insights that can inform public health initiatives and clinical practices tailored to the unique needs of African populations.

## Methodology

The protocol was registered in the International Prospective Register of Systematic Reviews (PROSPERO) under registration number CRD420251134616. This systematic review was conducted following the Joanna Briggs Institute (JBI) methodology for systematic reviews of prevalence, incidence, and risk/etiology [10], in line with the updated Preferred Reporting Items for Systematic Reviews and Meta-Analyses (PRISMA 2020) guidelines [11] (**S1** **and** **S2 Files**).

### Search strategy

Studies assessing the prevalence of VDD and genetic associations among African populations with T2DM were considered. All publicly available articles published up to June 11, 2025, were included, with no language restrictions. Relevant studies were identified through comprehensive searches of PubMed, Cochrane Library, ScienceDirect, Embase, Web of Science, and Google Scholar. Medical Subject Headings (MeSH) and keywords used included “Diabetes Mellitus, Type 2,” “Vitamin D,” “Genetic Polymorphism,” and “Africa,” with the names of individual African countries also incorporated. These terms were applied both independently and in combination using Boolean operators ‘AND’ and ‘OR’ to systematically identify eligible articles. Additionally, the reference lists of included studies were screened to identify further relevant publications, and we have contacted authors to identify studies with missing information. The database search was conducted from April 10 to June 11, 2025 (**S3 File**).

### Eligibility criteria

Studies were considered eligible if they were observational in design, including cross-sectional, case–control, or cohort studies, and assessed VDD among participants with T2DM. Eligible studies were required to report VDD prevalence, mean and standard deviation of vitamin D levels, or provide sufficient data to calculate mean differences (MD) or odds ratios (ORs). Studies examining genetic associations, particularly VDR gene variants, with T2DM or vitamin D status were also included. No restrictions were placed on language, and studies conducted in human populations of any age were considered.

Studies were excluded if they did not provide original data, such as reviews, editorials, or commentaries, or if they were case reports or series with fewer than 50 participants, as such small sample sizes were considered insufficient to generate reliable prevalence estimates. Studies lacking sufficient information to compute effect sizes (MD, standard deviations (SDs), ORs, or prevalence) were excluded, as were animal or in-vitro studies. In cases of duplicate or overlapping datasets, only the most complete or recent dataset was included in the analysis.

### Study selection

**Fig 1:**
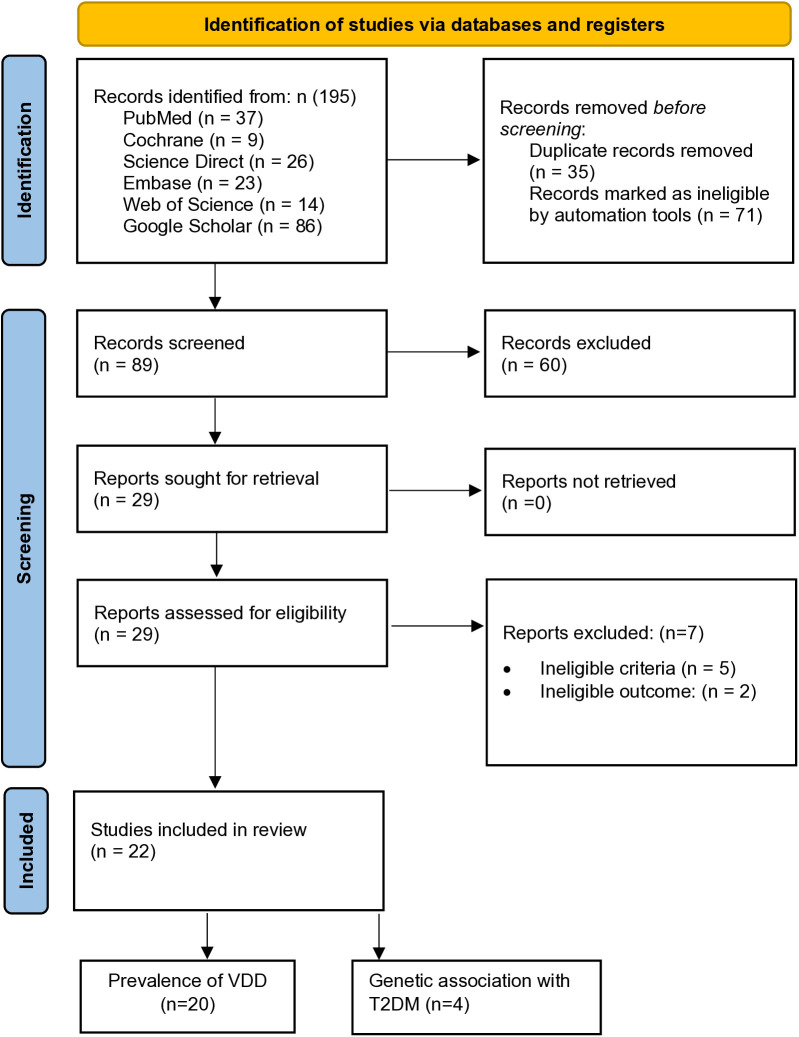
PRISMA flow diagram of search and study selection process.

All retrieved studies were initially screened for eligibility by assessing titles and abstracts. Full-text articles were then reviewed for inclusion based on pre-specified eligibility criteria. Two independent reviewers (EGA, SAH) conducted the screening and selection process, and any discrepancies were resolved through discussion or consultation with a third reviewer (EHG). A standardized screening form was used to ensure consistency in the assessment, and reasons for exclusion at the full-text stage were documented. The study selection process is summarized in a PRISMA flow diagram (**Fig 1**), illustrating the number of records identified, screened, assessed for eligibility, and included in the final analysis.

### Outcome measurements

The primary outcome of interest was the prevalence of VDD among the study populations. Secondary outcomes included the association of VDD with glycemic control, assessed through MD between vitamin D–deficient and sufficient groups, and the relationship between VDR gene variants and the risk of T2DM, assessed using ORs.

### Assessment of methodological quality

The methodological quality of the included studies was assessed using the Newcastle–Ottawa Scale (NOS), adapted for the specific study design (cross-sectional or case–control). For cross-sectional studies, the scale evaluated three domains: selection of participants, comparability of groups, and outcome assessment [12]. For case–control studies, the scale assessed selection of cases and controls, comparability of cases and controls, and ascertainment of exposure [13]. Each study was independently evaluated by two reviewers (EGA, SAH), with discrepancies resolved through discussion or consultation with a third reviewer (EHG). The NOS scores were recorded for each study, and a summary table was generated to provide an overview of study quality across all included studies (**S4 File**). This assessment included the non–peer-reviewed thesis from Algeria, which was retained for geographical representation.

### Data extraction

Data from eligible studies were independently extracted by two reviewers using a pre-designed Microsoft Excel sheet (EGA, SAH). Extracted information included: first author, publication year, country, study design, sample size, vitamin D measurement method, prevalence of vitamin D deficiency, glycemic control parameters (mean glucose or HbA1c), and VDR gene polymorphisms when reported. Any discrepancies between reviewers were resolved through discussion or consultation with a third reviewer (EHG). Data were cross-checked to ensure accuracy and completeness before statistical analyses. The full extracted dataset is provided as **S5 File**.

### Data synthesis and analysis

All statistical analyses were conducted using R version 4.3.1 (2023-06-16) with the meta and dplyr packages. In addition to calculating the pooled prevalence, we performed cumulative meta-analyses to examine how the overall estimate evolved as studies were added chronologically, allowing assessment of the temporal stability and accumulation of evidence.

For case–control studies, prevalence estimates were derived using only the T2DM (‘case’) group to ensure that the pooled estimates reflect the target population rather than the sampling ratio inherent to the study design.

To assess the impact of VDD on glycemic control, we used MD between deficient and sufficient groups. In studies reporting multiple subgroups or separate means and SDs, we combined them into a single summary measure using weighted calculations based on sample sizes. The association of VDR gene polymorphisms with T2DM was assessed using ORs with 95% confidence intervals (CIs). For meta-analytic pooling, random-effects models were applied to account for between-study variability, and statistical significance was considered at p < 0.05.

Heterogeneity between studies was assessed using Cochran’s Q test and quantified with the I^2^ statistic, with values of 25%, 50%, and 75% interpreted as low, moderate, and high heterogeneity, respectively [14]. To assess publication bias, we performed both Egger’s linear regression test and Begg’s rank correlation test. In addition, we applied the trim-and-fill method to estimate and adjust for potentially missing studies and funnel plot asymmetry, even when Egger’s and Begg’s tests indicated non-significant small-study effects. We conducted sensitivity analyses using a leave-one-out approach, where each study was sequentially omitted to evaluate its impact on the pooled estimates. A mixed-effects meta-regression was also conducted to explore source of heterogeneity.

The overall certainty of evidence for each outcome (prevalence of VDD, association with glycemic control, and genetic associations with T2DM) was assessed using the GRADE framework [15,16]. In this framework, evidence from randomized studies initially starts at high certainty, while evidence from observational studies starts at low certainty, and may be downgraded or upgraded based on predefined criteria. The five domains considered for potential downgrading included risk of bias, inconsistency, indirectness, imprecision, and publication bias, while factors such as large effect size, dose-response relationship, and residual confounding were considered for potential upgrading. Certainty ratings were determined by cumulatively evaluating these factors, with downgrading applied for limitations such as substantial heterogeneity or imprecision. Certainty of evidence was categorized as high, moderate, low, or very low.

Finally, results were visually summarized using forest plots to display pooled effect sizes and 95% CIs, funnel plots to examine potential publication bias, and tables to present descriptive characteristics, calculated outcomes, and study-level data. The R scripts used for all analyses are provided as **S6 File** to enhance transparency and reproducibility.

## Result

Out of 195 retrieved studies, 22 were eligible and included in the final analysis (Fig 1).

### Study selection and characteristics

A total of 22 studies published between 2014 and 2025 across 11 African countries were included, encompassing 3,447 participants. Except for two studies (9.1%) from Tunisia [17] and Ethiopia [18], which did not provide prevalence estimates, 20 studies (90.9%) reported VDD prevalence, which varied widely across settings. The highest prevalence was observed in Ghana (92.4%) [7] and Sudan (78.5%) [19], whereas the lowest rates were reported in Nigeria (3.3%) [6] and (5%) [20]. The included studies comprised 11 case-control studies (50%) and 11 cross-sectional studies (50%), with sample sizes ranging from 50 to 439 participants. Among the studies, most used a 20 ng/mL cut-off (n = 14); a few used other thresholds (n = 4), and three did not report a clear definition (Table 1).

**Table 1:** Characteristics of studies included in the systematic review on vitamin D deficiency in Africa.

Study	Year	Country	Design	Sample size	VDD Prevalence (%)	VDD_Cutoff
Abdelsadek et al. [21]	2018	Egypt	Case-control	60	73.3	28.3
Fondjo L. et al. [7]	2017	Ghana	Case-control	118	92.4	20
Batubo U. D. et al. [22]	2025	Nigeria	Case-control	166	77.3	20
Melake A. et al. [23]	2025	Ethiopia	Cross-sectional	306	49	20
Said J et al. [24]	2021	Kenya	Cross-sectional	124	71.1	20
Aljack et al. [19]	2019	Sudan	Cross-sectional	205	78.5	30
Karau et al. [25]	2019	Kenya	Cross-sectional	151	38.4	20
Fondjo L. [26]	2018	Ghana	Cross-sectional	192	61	20
Hassan A. [27]	2024	Sudan	Case-control	88	75	20
Raharinavalona A. [28]	2024	Madagascar	Cross-sectional	318	20.8	20
Erasmus R et al. [29]	2022	South Africa	Cross-sectional	204	50	20
Arhin-Aidoo F et al. [30]	2025	Ghana	Case-control	100	28	20
Mahjoubi I. et al. [17]	2016	Tunisia	Case–control	439	NA	NA
Melake A. et al. [18]	2025	Ethiopia	Case-control	70	NA	NA
Errouagui A et al. [31]	2014	Morocco	Case-control	176	40	20
El Gendy H I et al. [32]	2018	Egypt	Case-control	50	30	20
Adeleye J et al. [6]	2023	Nigeria	Case-control	120	3.3	20
Abbiyesuku M et al. [20]	2016	Nigeria	Case-control	80	5	20
Mostafa et al. [33]	2023	Egypt	Cross-sectional	100	60	30
Anyanwu A et al. [34]	2020	Nigeria	Cross-sectional	114	63.2	11.1
Safi S et al. [35]	2015	Morocco	Cross-sectional	211	52.3	10
Hasan A. et al. [36]	2022	Algeria	Cross-sectional	55	72.7	NA

VDD: Vitamin D deficiency.

### Pooled prevalence of VDD

**Fig 2:**
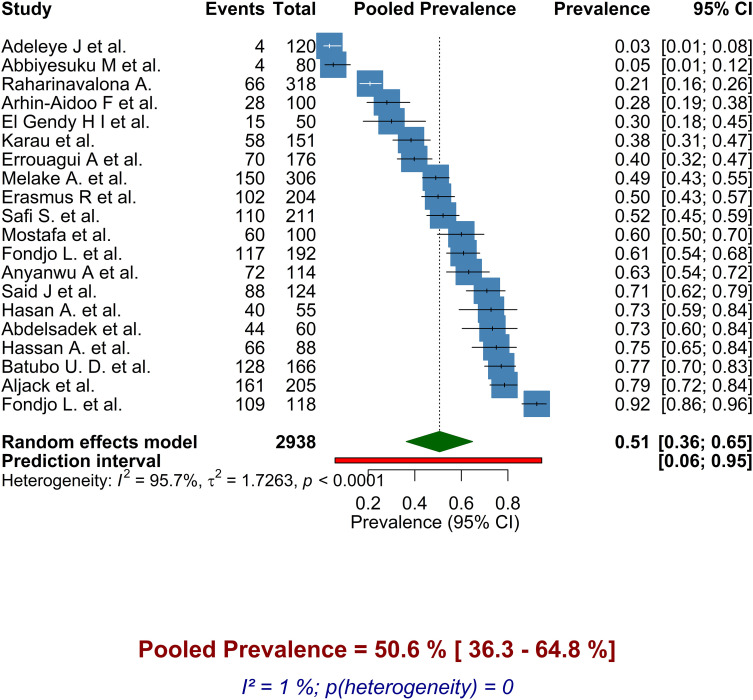
Forest plot showing the pooled prevalence of vitamin D deficiency among individuals with type 2 diabetes mellitus in Africa.

Among the 20 studies reporting VDD prevalence, comprising 2,938 participants, the pooled prevalence of VDD among individuals with T2DM in Africa was 51% (95% CI: 36–65%), with significant heterogeneity across studies (I^2^ = 95.7%, p < 0.001) (Fig 2).

### Cumulative meta-analysis

A cumulative meta-analysis, ordered by publication year, was performed to track the evolution of the pooled prevalence estimate as evidence accumulated. The initial estimates were highly volatile, with the point estimate ranging from 40% to 57% as the first 13 studies were incorporated. However, with the subsequent addition of larger studies from 2023 to 2025, the estimate converged and stabilized. The final pooled prevalence of 51% (95% CI: 49.0% to 53.0%), based on all 20 studies, is considered the most robust and precise estimate, reflecting the maturation of the evidence base (S1 Fig).

### Subgroup analyses by region

**Fig 3:**
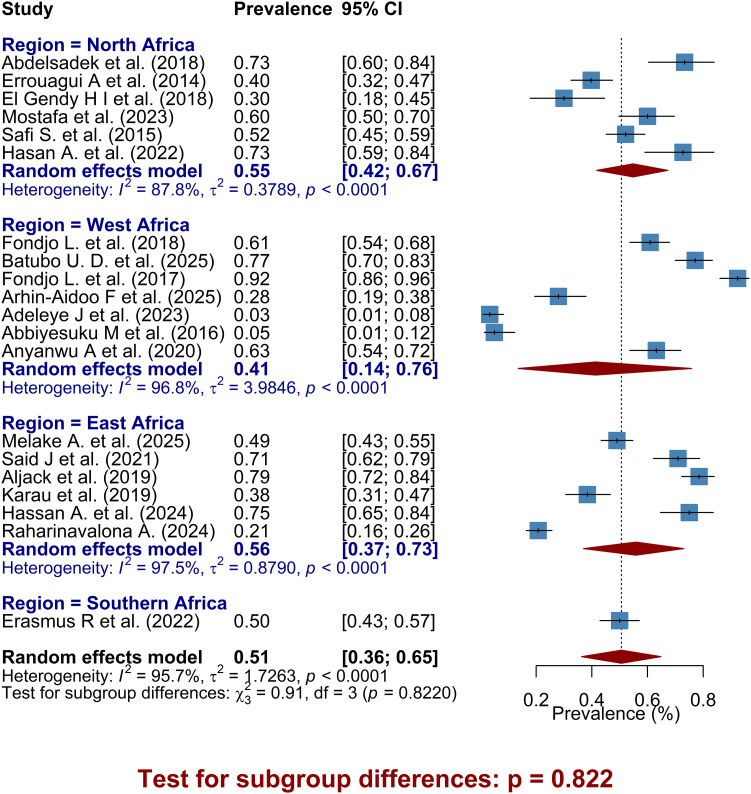
Subgroup forest plot of the pooled prevalence of vitamin D deficiency among individuals with type 2 diabetes in Africa, by region.

Subgroup analysis by region showed that the highest pooled prevalence, with substantial heterogeneity, was observed in East Africa at 56% (95% CI: 37–73%; I^2^ = 97.5%, p < 0.001) across six studies, followed by North Africa with a pooled prevalence of 55% (95% CI: 42–67%; I^2^ = 87.8%, p < 0.001) across six studies. Only one study was reported from South Africa. The chi-squared test for subgroup differences indicated no statistically significant difference between regions (χ² = 0.91, df = 3, p = 0.82) (Fig 3).

### Subgroup analyses by study design

**Fig 4:**
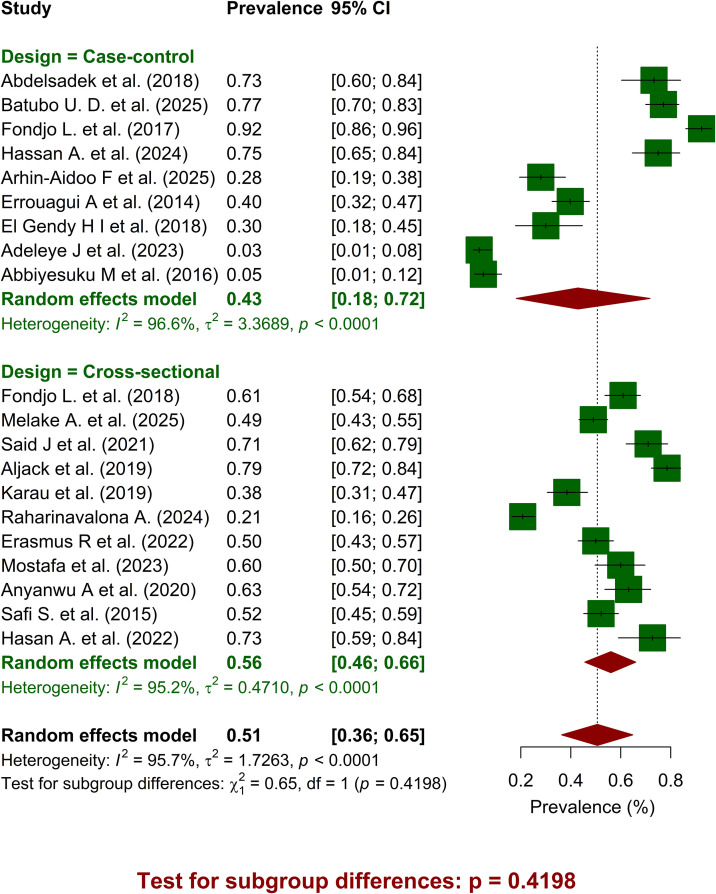
Subgroup forest plot of the pooled prevalence of vitamin D deficiency among individuals with type 2 diabetes in Africa, by study design.

We also conducted a subgroup analysis by study design. Among the included studies, 11 were cross-sectional and nine were case-control. The pooled prevalence of VDD was higher in cross-sectional studies at 56% (95% CI: 46–66%) with substantial heterogeneity (I^2^ = 95.2%, p < 0.001), compared to 43% (95% CI: 18–72%) in case-control studies, which also exhibited high heterogeneity (I^2^ = 96.6%, p < 0.001). The chi-squared test indicated no statistically significant difference between study designs (χ² = 0.65, df = 1, p = 0.42) (Fig 4).

### Subgroup analysis by vitamin D cutoff definition

**Fig 5:**
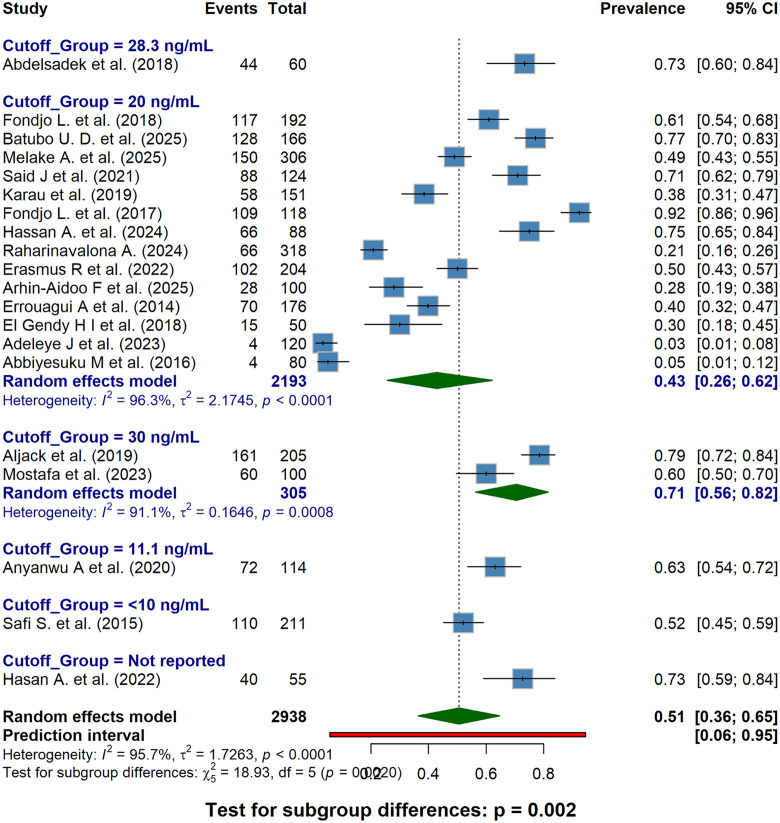
Subgroup forest plot showing pooled prevalence of vitamin D deficiency by cutoff definition. Test for subgroup differences: p = 0.002.

**Fig 6:**
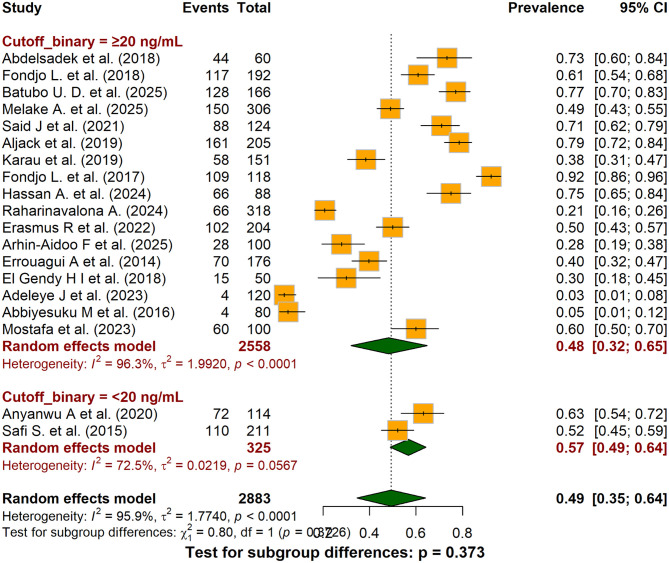
Subgroup forest plot comparing studies using <20 ng/mL vs ≥ 20 ng/mL cutoff definitions. Test for subgroup differences: p = 0.373.

Subgroup analysis by vitamin D cutoff definition revealed significant differences between groups (p = 0.002), with the highest pooled prevalence observed in studies using a cutoff of 30 ng/mL (71%, 95% CI: 56–82%) compared with those using 20 ng/mL (43%, 95% CI: 26–62%) (**Fig 5**). However, when dichotomized into clinically meaningful categories (<20 vs ≥ 20 ng/mL), no significant difference was observed (p = 0.373), indicating that the prevalence estimates were comparable when broader clinical thresholds were considered (**Fig 6**).

### Association between VDD and glycemic control

**Fig 7:**
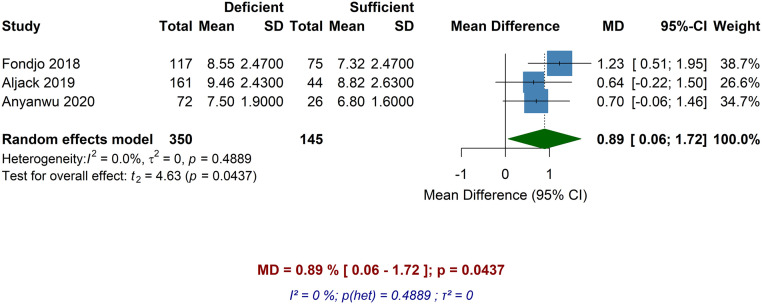
Forest plot showing the pooled mean difference in HbA1c (%) between vitamin D–deficient and sufficient individuals with type 2 diabetes in Africa.

The pooled analysis of three studies (n = 495) showed that vitamin D–deficient individuals had significantly higher HbA1c compared to vitamin D–sufficient individuals (MD = 0.89%, 95% CI: 0.06–1.72; p = 0.044; I^2^ = 0%, p = 0.49) (Fig 7).

### Association of VDR gene polymorphisms with T2DM

**Fig 8:**
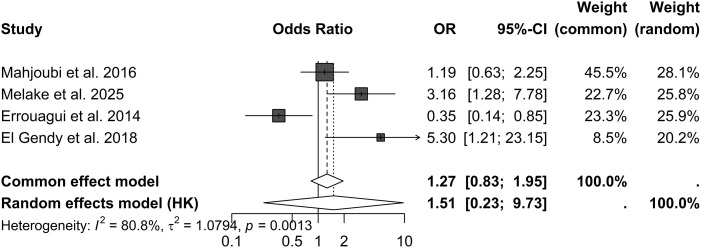
Pooled odds ratios for the association between VDR FokI genotype and risk of T2DM.

Across the four included studies (n = 735 participants), the distribution of VDR FokI genotypes varied considerably. The FF genotype ranged from 15.7% to 52.6%, the Ff genotype from 27% to 52%, and the ff genotype from 5.1% to 57.1%. The F allele frequency ranged from 29.3% to 73%, while the f allele ranged from 27% to 70.7%. These differences reflect population-specific genetic variation. Despite these variations, the pooled analysis of the association between FokI polymorphisms and T2DM did not show a statistically significant effect (pooled OR = 1.51, 95% CI: 0.23–9.73; I^2^ = 80.8%; p = 0.53) (Fig 8).

### Sensitivity analyses

A leave-one-out sensitivity analysis was performed to assess the robustness of the pooled VDD prevalence. Sequential omission of each study showed that the pooled prevalence remained stable, ranging from 47.5% to 54.8%, with consistently high heterogeneity (I^2^ = 94.5%–95.9%). The random-effects model yielded a pooled prevalence of 50.6% (95% CI: 36.3–64.8%), indicating that no single study disproportionately influenced the overall estimate (S2 Fig).

We additionally examined whether the varying definitions of VDD (<10–30 ng/mL; 63.6% using 20 ng/mL) influenced the findings. Sensitivity analysis showed no significant association between cutoff definitions and study characteristics (year: p = 0.746; design: p = 0.276; country: p = 0.605; sample size: p = 0.38), indicating that the variation in deficiency thresholds was randomly distributed and unlikely to systematically bias the pooled estimates.

### Publication bias

Assessment of publication bias using Egger’s (p = 0.879) and Begg’s (p = 0.846) tests revealed no significant small-study effects, and the trim-and-fill method did not impute any missing studies. Despite the absence of publication bias, heterogeneity remained high (I^2^ = 95.7%), reflecting substantial variation in VDD prevalence across the included studies (**S3 Fig**).

### Meta-regression analysis

A mixed-effects meta-regression was conducted to explore whether study-level characteristics, including publication year and sample size, explained heterogeneity in VDD prevalence across 20 studies. Residual heterogeneity remained high (τ² = 1.718; I^2^ = 97.9%), and the test for moderators was not significant (QM = 0.06, df = 2, p = 0.971). We also examined the influence of vitamin D cutoff definitions using meta-regression, treating the cutoff value as a continuous variable; however, this factor did not significantly explain heterogeneity in prevalence estimates (p = 0.473). Together, these findings indicate that publication year, sample size, and cutoff definitions did not account for the observed variability, suggesting that the substantial heterogeneity is likely driven by other unmeasured methodological or population-level factors.

### Risk of bias (study quality assessment)

The methodological quality of the included studies was further appraised using the NOS. Overall, 11 studies (50.0%) were rated as high quality (≥7 stars), 8 (36.4%) as moderate quality (5–6 stars), and 3 (13.6%) as low quality (≤4 stars) (**S4 File**).

### Certainty of evidence

The certainty of evidence for the pooled prevalence of vitamin D deficiency was moderate, downgraded for substantial heterogeneity (I^2^ = 95.7%). Evidence supporting the association between vitamin D deficiency and higher HbA1c was low, due to imprecision and limited number of contributing studies. Evidence regarding VDR FokI polymorphisms and T2DM risk was very low, reflecting small sample sizes, wide confidence intervals, and inconsistency across studies. These assessments indicate that while the prevalence estimates are reasonably reliable, the evidence for clinical and genetic associations remains limited and should be interpreted with caution.

## Discussion

This systematic review and meta-analysis found a high pooled prevalence of VDD among African individuals with T2DM at 51% (95% CI: 36–65%), accompanied by significant heterogeneity across studies. Subgroup analyses indicated slightly higher prevalence in East Africa (56%) and North Africa (55%), although differences were not statistically significant. Vitamin D deficiency was also significantly associated with poorer glycemic control, reflected by a higher mean HbA1c of 0.89% (95% CI: 0.06–1.72; p = 0.044; I^2^ = 0%, p = 0.49) in deficient individuals. However, the pooled analysis did not find a significant association between vitamin D receptor (VDR) FokI polymorphisms and T2DM risk.

The high prevalence of VDD is consistent with global evidence showing high rates of VDD among people with diabetes, despite Africa’s abundant sunlight [37]. This apparent paradox can be explained by several interconnected factors. First, darker skin pigmentation, prevalent across African populations, reduces cutaneous vitamin D synthesis due to higher melanin content, which acts as a natural sunscreen by absorbing UVB radiation [38]. Second, rapid urbanization across the continent has shifted populations toward indoor occupations and housing, significantly limiting daily sun exposure [34]. Third, traditional African diets are generally low in vitamin D-rich foods such as fatty fish and fortified dairy products, and widespread food fortification remains limited in most African countries [4]. Cultural practices such as clothing that limits sun exposure further contribute to reduced synthesis [38].

These combined biological, behavioral, and nutritional factors likely drive the high VDD burden despite favorable climatic conditions. In Kenya, for example, the prevalence of hypovitaminosis D among T2DM patients was reported as 71.1%, with overall insufficiency rates exceeding 90% [24]. Similarly, a Ghanaian case–control study found a prevalence of 92.4% [7], whereas lower prevalence was noted in Nigerian cohorts, where only 3.3% of participants were deficient [6]. Differences in assay methods, cutoffs for deficiency, and study designs may also contribute to the observed variations across settings.

In comparison with previous systematic reviews, our findings provide a more comprehensive representation of the African context. A recent global systematic review that assessed VDD among individuals with T2DM reported, in subgroup analysis, that African nations contributed only five studies involving 658 participants, with a pooled prevalence of 70.9% (95% CI: 52.8–88.9) based on studies published up to 31 January 2023 which we also included them in the S6 File for comparison [39]. These studies are also included in our S5 File for comparison. While informative, this analysis substantially underrepresented African populations, as it omitted at least 10 eligible studies that were available before that date [6,20,26,29,31–36]. In contrast, our review identified and analyzed 15 studies among 1, 960 participants conducted before January 2023, in addition to more recent publications, thereby offering a more robust and regionally inclusive estimate of VDD burden in Africa. This highlights the importance of region-focused evidence syntheses, since global reviews may not fully capture the heterogeneity and scale of the problem across African settings.

The significant association between VDD and poorer glycemic control supports vitamin D’s role in enhancing insulin secretion, modulating beta-cell function, and reducing inflammation linked to insulin resistance [40,41]. This relationship is consistent with studies from Nigeria, where lower vitamin D levels were correlated with higher insulin resistance measured by HOMA-IR [6], and with other African cohorts reporting higher HbA1c and fasting plasma glucose among VDD subjects [34]. Clinically, VDD may exacerbate hyperglycemia and related complications [42], highlighting the importance of vitamin D assessment in diabetes care in Africa. However, this finding should be interpreted with caution, as it is based on a limited number of studies with relatively wide confidence intervals. In addition, the included studies did not consistently adjust for key confounders such as diabetes duration, medication use, BMI, and renal function, and therefore the observed association should be considered hypothesis-generating.

The observed 0.89% mean difference in HbA1c between vitamin D-deficient and sufficient individuals is clinically meaningful. For context, this magnitude of reduction is comparable to that achieved with commonly used glucose-lowering agents such as metformin (approximately 1.0–1.5%) and sulfonylureas (approximately 1.0–2.0%) [43]. Furthermore, large-scale clinical trials have demonstrated that each 1% reduction in HbA1c is associated with approximately 21% reduction in diabetes-related complications, including microvascular outcomes [44]. While these comparisons are instructive, they should be interpreted cautiously given the observational nature of our evidence and the potential for residual confounding. Nevertheless, the magnitude of association suggests that if causality is confirmed through rigorous interventional trials, optimizing vitamin D status could have substantial clinical benefits for glycemic management and complication risk reduction in African populations with T2DM.

Regarding genetic factors, our pooled analysis found no significant association between VDR FokI polymorphisms and T2DM risk in African populations. However, results varied across individual studies. For instance, Ethiopian data suggested that the FokI “ff” genotype and “f” allele were associated with increased susceptibility to T2DM [18], whereas Tunisian research reported no significant associations [17]. These inconsistencies may stem from Africa’s genetic diversity, with variations in allele frequency and linkage disequilibrium across populations, as well as differences in study size and methodology. Meta-analyses in Asian populations, by contrast, have shown clearer associations between VDR variants and T2DM [45], underscoring the importance of population-specific genomic research in Africa. This suggests VDR polymorphisms alone may not substantially influence T2DM risk, pointing to gene-environment interactions and other genetic factors requiring further research.

Substantial heterogeneity remained unexplained by publication year or sample size in the meta-regression, suggesting that other unmeasured factors likely contributed to the observed variability. These may include seasonal differences in sampling, degree of urbanization, dietary patterns, sun exposure behaviors, assay methods, and varying definitions of deficiency (e.g., < 20 ng/mL vs. higher thresholds). Population characteristics such as obesity prevalence and comorbid conditions may also have played a role. Nevertheless, sensitivity analyses demonstrated that no single study disproportionately influenced the pooled prevalence, underscoring the robustness of our overall estimate.

### Strength and limitation

This review is the first to comprehensively synthesize evidence on the prevalence of VDD and its genetic associations with T2DM across diverse African settings. We employed a pre-registered protocol, followed rigorous JBI and PRISMA 2020 guidelines, and searched multiple databases without language restrictions, thereby minimizing selection bias. The inclusion of both prevalence and genetic studies allowed us to provide a multidimensional perspective on the role of vitamin D in diabetes. In addition, cumulative and sensitivity analyses strengthened the reliability of the pooled estimates, and subgroup/meta-regression analyses provided insight into sources of heterogeneity.

Despite these strengths, several limitations should be acknowledged. First, significant heterogeneity persisted even after subgroup and meta-regression analyses, reflecting variations in study populations, laboratory assays, environmental exposures, and study design across Africa. The definition of vitamin D deficiency also varied considerably, with cutoffs ranging from <10–30 ng/mL. Although sensitivity analyses showed no significant association between cutoff definitions and study characteristics, this variation remains an important source of clinical heterogeneity. Second, the very high heterogeneity observed limits the generalizability of the pooled prevalence estimates, suggesting caution when applying these findings to regional or clinical settings. Third, the number of genetic studies was small, with inconsistent reporting of allele distributions, limiting the robustness of conclusions regarding VDR polymorphisms. Fourth, most included studies were observational and cross-sectional, precluding causal inference between vitamin D status and glycemic outcomes. Fifth, meta-regression analyses were limited to publication year, sample size, and vitamin D cutoff thresholds, as other potentially relevant moderators (e.g., assay type, study setting, or population characteristics) could not be formally assessed due to incomplete reporting in the primary studies. Seasonal variation, in particular, may have influenced measured vitamin D levels and remains an unaccounted confounder. Sixth, publication bias could not be entirely excluded, as grey literature and unpublished studies may have been missed despite comprehensive searching. Finally, regional representation was uneven, with some African countries lacking data, which may limit generalizability across the continent.

## Conclusion

This systematic review and meta-analysis shows that VDD is highly prevalent among individuals with T2DM in Africa, affecting about half of this population. Despite abundant sunlight, deficiency remains widespread and is associated with poorer glycemic control, highlighting vitamin D as a potentially modifiable risk factor in diabetes management. The pooled analysis did not demonstrate a consistent association between VDR FokI polymorphisms and diabetes risk; however, given the small number of genetic studies and the extensive genetic diversity across African populations, these findings should be considered hypothesis-generating and underscore the need for larger, population-specific, multi-country genetic studies.

These findings underscore the need for high-quality interventional trials to determine whether improving vitamin D status enhances glycemic outcomes in this population; until such evidence is available, routine screening or supplementation recommendations remain premature. Future research should prioritize longitudinal and interventional studies, alongside pan-African genomic investigations, to better define causal pathways and inform evidence-based public health strategies.

## Supporting information

S1 File PRISMA 2020 checklist for reporting the findings of the systematic review on vitamin D deficiency prevalence among African populations with type 2 diabetes. (PDF)

S2 File PRISMA 2020 checklist for reporting the findings of the systematic review on vitamin D deficiency prevalence and genetic associations among African populations with type 2 diabetes. (PDF)

S3 File Search strategy of the systematic review and meta-analysis on the on vitamin D deficiency prevalence and genetic associations among African populations with type 2 diabetes. (PDF)

S4 File Risk of bias of included studies the systematic review and meta-analysis on the on vitamin D deficiency prevalence and genetic associations among African populations with type 2 diabetes. (PDF)

S5 File Extracted data from included studies on vitamin D deficiency and VDR polymorphisms in African populations with type 2 diabetes. (XLSX)

S6 File R scripts for meta-analysis, sensitivity analyses, and data visualization for vitamin D deficiency and VDR polymorphism studies in African populations with type 2 diabetes. (R)

S1 Fig Cumulative meta-analysis showing the temporal trend and stabilization of VDD prevalence across included studies. (TIF)

S2 Fig Leave-One-Out sensitivity analysis of pooled VDD prevalence. (TIF)

S3 Fig Funnel plot assessing publication bias in studies reporting VDD prevalence. (TIF)
