# Supplementary material for: Hidden deficiency under bright skies: Vitamin D prevalence and genetic associations in African Type 2 diabetes: A systematic review and meta-analysis
Source: PLoS One. 2026 Jul 24;21(7):e0354518. doi: 10.1371/journal.pone.0354518 (PMC13399315; doi:10.1371/journal.pone.0354518)
Supplement: S3 File — (PDF) [file pone.0354518.s003.pdf]

Hidden Deficiency under Bright Skies: Vitamin D Prevalence and Genetic Associations in African Type 2 Diabetes: A Systematic Review and Meta-Analysis

Searching Path

- PubMed (n=37)
- Cochrane Library (n=9)
- Science Direct (n=26)
- Embase (n=23)
- Web of Science (n=14)
- Google Scholar (n=86)

Main Path: “Diabetes Mellitus, Type 2” AND “Vitamin D” AND “Polymorphism, Genetic” AND “Africa” – we have used each African countries

PubMed

**Population (Po)**<br>**Type 2 DM patients**	**Exposure (E)**<br>**Vitamin D Status**	**Context (Africa)**	**Outcome (O)**<br>**Genetic Associations**
“Diabetes Mellitus, Type 2” [Mesh term]	"Vitamin D"[MeSH Terms]	"Africa"[Mesh]	“Polymorphism, Genetic” [Mesh]
"Type 2 Diabetes"[Title/Abstract]	"Vitamin D"[Title/Abstract]	"Africa South of the Sahara"[Mesh]	“Polymorphisms” [Title/Abstract]
"Type II Diabetes"[Title/Abstract]	"25-hydroxyvitamin D"[Title/Abstract]	"Africa, Northern"[Mesh]	"FokI"[Title/Abstract]
"Diabetes Mellitus Type 2"[Title/Abstract]	"25 hydroxyvitamin D"[Title/Abstract]	"Africa, Eastern"[Mesh]	"Fok I"[Title/Abstract]
"Non-insulin dependent diabetes"[Title/Abstract]	"25(OH)D"[Title/Abstract]	"Africa, Western"[Mesh]	"BsmI"[Title/Abstract]

"NIDDM"[Title/Abstract]	"cholecalciferol"[Title/Abstract]	"Africa, Southern"[Mesh]	"ApaI"[Title/Abstract]
"T2DM"[Title/Abstract]	"ergocalciferol"[Title/Abstract]	"ALGERIA"[Title/Abstract]	"TaqI"[Title/Abstract]
"Type 2 Diabetes"[Title/Abstract]	"vitamin D status"[Title/Abstract]	"ANGOLA"[Title/Abstract]	"genetic variant"[Title/Abstract]
"Type II Diabetes"[Title/Abstract]	"vitamin D deficiency"[Title/Abstract]	"BENIN"[Title/Abstract]	
"Diabetes Mellitus Type 2"[Title/Abstract]	"hypovitaminosis D"[Title/Abstract]	"BOTSWANA"[Title/Abstract]	
"Non-insulin dependent diabetes"[Title/Abstract]	"Vitamin D Deficiency"[Mesh]	"BURKINA FASO"[Title/Abstract]	
		"BURUNDI"[Title/Abstract]	
		"CABO VERDE"[Title/Abstract]	
		"CAMEROON"[Title/Abstract]	
		"CENTRAL AFRICAN REPUBLIC"[Title/Abstract]	
		"CHAD"[Title/Abstract]	
		"COMOROS"[Title/Abstract]	
		"CONGO"[Title/Abstract]	
		"COTE D'IVOIRE"[Title/Abstract]	
		"DEMOCRATIC REPUBLIC OF THE CONGO"[Title/Abstract]	
		"DJIBOUTI"[Title/Abstract]	
		"EGYPT"[Title/Abstract]	
		"EQUATORIAL GUINEA"[Title/Abstract]	
		"ERITREA"[Title/Abstract]	
		"ESWATINI"[Title/Abstract]	
		"ETHIOPIA"[Title/Abstract]	
		"GABON"[Title/Abstract]	
		"GAMBIA"[Title/Abstract]	
		"GHANA"[Title/Abstract]	

		"GUINEA"[Title/Abstract]	
		"GUINEA-BISSAU"[Title/Abstract]	
		"KENYA"[Title/Abstract]	
		"LESOTHO"[Title/Abstract]	
		"LIBERIA"[Title/Abstract]	
		"LIBYA"[Title/Abstract]	
		"MADAGASCAR"[Title/Abstract]	
		"MALAWI"[Title/Abstract]	
		"MALI"[Title/Abstract]	
		"MAURITANIA"[Title/Abstract]	
		"MAURITIUS"[Title/Abstract]	
		"MOROCCO"[Title/Abstract]	
		"MOZAMBIQUE"[Title/Abstract]	
		"NAMIBIA"[Title/Abstract]	
		"NIGER"[Title/Abstract]	
		"NIGERIA"[Title/Abstract]	
		"RWANDA"[Title/Abstract]	
		"SAO TOME AND PRINCIPE"[Title/Abstract]	
		"SENEGAL"[Title/Abstract]	
		"SEYCHELLES"[Title/Abstract]	
		"SIERRA LEONE"[Title/Abstract]	
		"SOMALIA"[Title/Abstract]	
		"SOUTH AFRICA"[Title/Abstract]	
		"SOUTH SUDAN"[Title/Abstract]	
		"SUDAN"[Title/Abstract]	
		"TANZANIA"[Title/Abstract]	
		"TOGO"[Title/Abstract]	
		"TUNISIA"[Title/Abstract]	
		"UGANDA"[Title/Abstract]	

		"ZAMBIA"[Title/Abstract]	
		"ZIMBABWE"[Title/Abstract]	
For Prevalence			
("Vitamin D"[Title/Abstract] OR "25-hydroxyvitamin D"[Title/Abstract] OR "25 hydroxyvitamin D"[Title/Abstract] OR "25(OH)D"[Title/Abstract] OR "cholecalciferol"[Title/Abstract] OR "ergocalciferol"[Title/Abstract] OR "vitamin D status"[Title/Abstract] OR "vitamin D deficiency"[Title/Abstract] OR "hypovitaminosis D"[Title/Abstract] OR "Vitamin D Deficiency"[Mesh] OR "Vitamin D"[Mesh]) AND ("Type 2 Diabetes"[Title/Abstract] OR "Type II Diabetes"[Title/Abstract] OR "Diabetes Mellitus Type 2"[Title/Abstract] OR "Non-insulin dependent diabetes"[Title/Abstract] OR "NIDDM"[Title/Abstract] OR "T2DM"[Title/Abstract] OR "Diabetes Mellitus, Type 2"[Mesh]) AND ("ALGERIA"[Title/Abstract] OR "ANGOLA"[Title/Abstract] OR "BENIN"[Title/Abstract] OR "BOTSWANA"[Title/Abstract] OR "BURKINA FASO"[Title/Abstract] OR "BURUNDI"[Title/Abstract] OR "CABO VERDE"[Title/Abstract] OR "CAMEROON"[Title/Abstract] OR "CENTRAL AFRICAN REPUBLIC"[Title/Abstract] OR "CHAD"[Title/Abstract] OR "COMOROS"[Title/Abstract] OR "CONGO"[Title/Abstract] OR "COTE D'IVOIRE"[Title/Abstract] OR "DEMOCRATIC REPUBLIC OF THE CONGO"[Title/Abstract] OR "DJIBOUTI"[Title/Abstract] OR "EGYPT"[Title/Abstract] OR "EQUATORIAL GUINEA"[Title/Abstract] OR "ERITREA"[Title/Abstract] OR "ESWATINI"[Title/Abstract] OR "ETHIOPIA"[Title/Abstract] OR "GABON"[Title/Abstract] OR "GAMBIA"[Title/Abstract] OR "GHANA"[Title/Abstract] OR "GUINEA"[Title/Abstract] OR "GUINEA-BISSAU"[Title/Abstract] OR "KENYA"[Title/Abstract] OR "LESOTHO"[Title/Abstract] OR "LIBERIA"[Title/Abstract] OR "LIBYA"[Title/Abstract] OR "MADAGASCAR"[Title/Abstract] OR "MALAWI"[Title/Abstract] OR "MALI"[Title/Abstract] OR "MAURITANIA"[Title/Abstract] OR "MAURITIUS"[Title/Abstract] OR "MOROCCO"[Title/Abstract] OR "MOZAMBIQUE"[Title/Abstract] OR "NAMIBIA"[Title/Abstract] OR "NIGER"[Title/Abstract] OR "NIGERIA"[Title/Abstract] OR "RWANDA"[Title/Abstract] OR "SAO TOME AND PRINCIPE"[Title/Abstract] OR "SENEGAL"[Title/Abstract] OR "SEYCHELLES"[Title/Abstract] OR "SIERRA LEONE"[Title/Abstract] OR "SOMALIA"[Title/Abstract] OR "SOUTH AFRICA"[Title/Abstract] OR "SOUTH SUDAN"[Title/Abstract] OR "SUDAN"[Title/Abstract] OR "TANZANIA"[Title/Abstract] OR "TOGO"[Title/Abstract] OR "TUNISIA"[Title/Abstract] OR "UGANDA"[Title/Abstract] OR "ZAMBIA"[Title/Abstract] OR "ZIMBABWE"[Title/Abstract] OR "Africa"[Mesh] OR "Africa South of the Sahara"[Mesh] OR "Africa, Northern"[Mesh] OR "Africa, Eastern"[Mesh] OR "Africa, Western"[Mesh] OR "Africa, Southern"[Mesh])			
For Genetic Association			
("Vitamin D"[Title/Abstract] OR "25-hydroxyvitamin D"[Title/Abstract] OR "25 hydroxyvitamin D"[Title/Abstract] OR "25(OH)D"[Title/Abstract] OR "cholecalciferol"[Title/Abstract] OR "ergocalciferol"[Title/Abstract] OR "vitamin D status"[Title/Abstract] OR "vitamin D deficiency"[Title/Abstract] OR "hypovitaminosis D"[Title/Abstract] OR "Vitamin D Deficiency"[Mesh] OR "Vitamin D"[Mesh]) AND ("Type 2 Diabetes"[Title/Abstract] OR "Type II Diabetes"[Title/Abstract] OR			

"Diabetes Mellitus Type 2"[Title/Abstract] OR "Non-insulin dependent diabetes"[Title/Abstract] OR "NIDDM"[Title/Abstract] OR "T2DM"[Title/Abstract] OR "Diabetes Mellitus, Type 2"[Mesh]) AND ("ALGERIA"[Title/Abstract] OR "ANGOLA"[Title/Abstract] OR "BENIN"[Title/Abstract] OR "BOTSWANA"[Title/Abstract] OR "BURKINA FASO"[Title/Abstract] OR "BURUNDI"[Title/Abstract] OR "CABO VERDE"[Title/Abstract] OR "CAMEROON"[Title/Abstract] OR "CENTRAL AFRICAN REPUBLIC"[Title/Abstract] OR "CHAD"[Title/Abstract] OR "COMOROS"[Title/Abstract] OR "CONGO"[Title/Abstract] OR "COTE D'IVOIRE"[Title/Abstract] OR "DEMOCRATIC REPUBLIC OF THE CONGO"[Title/Abstract] OR "DJIBOUTI"[Title/Abstract] OR "EGYPT"[Title/Abstract] OR "EQUATORIAL GUINEA"[Title/Abstract] OR "ERITREA"[Title/Abstract] OR "ESWATINI"[Title/Abstract] OR "ETHIOPIA"[Title/Abstract] OR "GABON"[Title/Abstract] OR "GAMBIA"[Title/Abstract] OR "GHANA"[Title/Abstract] OR "GUINEA"[Title/Abstract] OR "GUINEA-BISSAU"[Title/Abstract] OR "KENYA"[Title/Abstract] OR "LESOTHO"[Title/Abstract] OR "LIBERIA"[Title/Abstract] OR "LIBYA"[Title/Abstract] OR "MADAGASCAR"[Title/Abstract] OR "MALAWI"[Title/Abstract] OR "MALI"[Title/Abstract] OR "MAURITANIA"[Title/Abstract] OR "MAURITIUS"[Title/Abstract] OR "MOROCCO"[Title/Abstract] OR "MOZAMBIQUE"[Title/Abstract] OR "NAMIBIA"[Title/Abstract] OR "NIGER"[Title/Abstract] OR "NIGERIA"[Title/Abstract] OR "RWANDA"[Title/Abstract] OR "SAO TOME AND PRINCIPE"[Title/Abstract] OR "SENEGAL"[Title/Abstract] OR "SEYCHELLES"[Title/Abstract] OR "SIERRA LEONE"[Title/Abstract] OR "SOMALIA"[Title/Abstract] OR "SOUTH AFRICA"[Title/Abstract] OR "SOUTH SUDAN"[Title/Abstract] OR "SUDAN"[Title/Abstract] OR "TANZANIA"[Title/Abstract] OR "TOGO"[Title/Abstract] OR "TUNISIA"[Title/Abstract] OR "UGANDA"[Title/Abstract] OR "ZAMBIA"[Title/Abstract] OR "ZIMBABWE"[Title/Abstract] OR "Africa"[Mesh] OR "Africa South of the Sahara"[Mesh] OR "Africa, Northern"[Mesh] OR "Africa, Eastern"[Mesh] OR "Africa, Western"[Mesh] OR "Africa, Southern"[Mesh]) AND “Polymorphism, Genetic” [Mesh] OR “Polymorphisms” [Title/Abstract] OR "FokI"[Title/Abstract] OR "Fok I"[Title/Abstract] OR "BsmI"[Title/Abstract] OR "ApaI"[Title/Abstract] OR "TaqI"[Title/Abstract] OR "genetic variant"[Title/Abstract]

Refined studies

Activities	PubMed	Cochrane	Science Direct	Embase	Web of Science	Google Scholar	Total
Exported data to Endnote after filtration	37	9	26	23	14	86	**195**
