# Supplementary material for: Hidden deficiency under bright skies: Vitamin D prevalence and genetic associations in African Type 2 diabetes: A systematic review and meta-analysis
Source: PLoS One. 2026 Jul 24;21(7):e0354518. doi: 10.1371/journal.pone.0354518 (PMC13399315; doi:10.1371/journal.pone.0354518)
Supplement: S4 File — (PDF) [file pone.0354518.s004.pdf]

<u>Newcastle–Ottawa Scale (Case-Sectional Study) – individual studies assessment</u>

1. Mahjoubi et al., 2016

Reference: Mahjoubi I, Kallel A, Sbaï MH, Ftouhi B, ben Halima M, Jemaa Z, et al. Lack of association between FokI polymorphism in vitamin D receptor gene (VDR) & type 2 diabetes mellitus in the Tunisian population. Indian J Med Res. 2016;144(1):46–51.

Selection (max 4 stars)

1. **Is the case definition adequate?**
   ★ 1/1
   Justification: Cases were defined as patients with T2DM diagnosed according to WHO criteria (objective biochemical criteria) and enrolled from the endocrinology department. This is an objective, standard diagnostic definition.
2. **Representativeness of the cases**
   No star.
   Justification: Cases were hospital patients enrolled at the Department of Endocrinology (Jan 2007–Jul 2009). The paper does not state that cases were a consecutive or population-based sample, so there is potential for selection bias (hospital-based series).
3. **Selection of controls**
   No star.
   Justification: Controls were recruited from individuals who "underwent a medical checkup in our hospital" i.e., hospital/check-up controls, not community controls. This increases chance of selection bias versus community-derived controls.
4. **Definition of controls**
   ★ 1/1
   Justification: Controls were excluded if fasting blood glucose >100 mg/dL or if they had a family history of diabetes so controls were defined as without the outcome.

Selection subtotal: 2 / 4 stars.

Comparability (max 2 stars)

5. **Comparability of cases and controls on design/analysis**
   0 / 2 stars.
   Justification: Although logistic regression was used to estimate ORs, the paper does not report adjusted analyses (no clear statement of which covariates were included). Importantly, cases and controls differed significantly on key covariates (age, BMI, dyslipidaemia, hypertension, smoking); these are potential confounders that were not shown to be controlled for in the genetic association analyses. Therefore no star(s) for comparability.

Exposure (max 3 stars)

6. **Ascertainment of exposure**
   ★ 1/1
   Justification: Genotyping of the FokI (rs2228570) polymorphism was done by PCR-RFLP in the laboratory (description of primers, enzyme digestion); laboratory genotyping is an objective measure.
7. Same method of ascertainment for cases and controls
   ★ 1/1
   Justification: All samples (cases and controls) were genotyped using the same PCR-RFLP protocol; genotype calls were made independently by two technicians and 20% of samples were re-genotyped with 100% concordance; consistent methods across groups.
8. **Non-response rate**
   No star.
   Justification: The study does not describe participation/non-response rates or whether response rates were similar for cases and controls, this item is not reported.

Exposure subtotal: 2 / 3 stars.

Total = 4/9

2. Melake A. et al., 2025

Reference: Melake A, Nakachew E. Association between vitamin D receptor FokI gene polymorphism and risk of type 2 diabetes mellitus in the Ethiopian population. Sci Rep. 2025;15(1):27248.

Selection (max 4 stars)

1. **Is the case definition adequate?**
   ★ 1/1
   Justification: Cases were defined using objective biochemical criteria (FBG ≥126 mg/dL, RBG ≥200 mg/dL, or treatment with hypoglycemics/insulin), i.e., standard diagnostic thresholds for T2DM.
2. **Representativeness of the cases**
   ★ 1/1
   Justification: This was a hospital-based study with the source population defined as all chronic follow-up clinic (CFC) patients; participants were chosen from registered patients using a table of random numbers (TRN), which indicates a representative sampling approach from the clinic population.
3. **Selection of controls**
   ★ 1/1
   Justification: Controls are described as age- and sex-matched *nondiabetic healthy volunteers* from the same socioeconomic position and geographic region (not described as 'hospital check-up' controls). The description aligns with community/healthy control selection rather than convenience hospital check-ups. (Paper text: "Any nondiabetic, healthy volunteers … served as the study's controls.").
4. **Definition of controls**
   ★ 1/1
   Justification: Controls were screened (normal blood glucose) and individuals with long-term NCDs or persistent infections were excluded, i.e., defined as without the outcome.

Selection subtotal: 4 / 4 stars.

Comparability (max 2 stars)

5. **Comparability of cases and controls on the basis of design or analysis**
   ★ 1 / 2.
   Justification: The study used *age- and sex-matching* (important confounders) and reports similar distributions for age and sex between groups. Logistic regression was used to estimate associations, but the paper does **not** present multivariable adjusted estimates that explicitly control for additional potential confounders (e.g., BMI, hypertension); therefore only the single star for controlling the most important factors via matching.

Exposure (max 3 stars)

6. **Ascertainment of exposure**
   ★ 1/1
   Justification: Genotypes were determined by laboratory PCR-RFLP (DNA extraction, spectrophotometry for purity, PCR, restriction digest, gel electrophoresis); objective molecular ascertainment.
7. **Same method of ascertainment for cases and controls**
   ★ 1/1
   Justification: All samples (cases and controls) underwent the same DNA extraction and PCR-RFLP workflow described in Methods.
8. **Non-response rate**
   No star.
   Justification: The paper does not report participation / non-response rates or whether rates were similar between cases and controls (no numbers approached vs enrolled).

Exposure subtotal: 2 / 3 stars.

Total = 7/9

3. Fondjo L. et al., 2017

Reference: Fondjo LA, Owiredu WKBA, Sakyi SA, Laing EF, Adotey-Kwofie MA, Antoh EO, et al. Vitamin D status and its association with insulin resistance among type 2 diabetics: A case-control study in Ghana. PLoS One. 2017;12(4):e0175388.

Selection (max 4 stars)

1. **Is the case definition adequate?**
   ★ 1/1
   Justification*:* Cases were clinically diagnosed type 2 diabetes mellitus (T2DM) patients attending the Diabetic Clinic at Nkawie Government Hospital. Diagnosis was based on established clinical criteria, ensuring adequate case definition.
2. **Representativeness of the cases**
   ★ 1/1
   Justification*:* Participants were selected from a hospital-based diabetic clinic, representing a specific patient population. While hospital-based, the selection appears systematic and relevant to the study's objectives.
3. **Selection of controls**
   ★ 1/1
   Justification*:* Controls were 100 healthy non-diabetic individuals from the Nkawie district, matched by age and sex. This approach minimizes selection bias and provides a suitable comparison group.
4. **Definition of controls**
   ★ 1/1
   Justification*:* Controls were defined as healthy non-diabetic individuals with no history of diabetes, ensuring they are free from the outcome of interest.

Selection Subtotal: 4/4 stars

Comparability (max 2 stars)

5. **Comparability of cases and controls on the basis of design or analysis**
   ★ 1/2
   Justification*:* The study matched cases and controls by age and sex. However, it does not report adjustments for other

potential confounders such as BMI, physical activity, or dietary factors, which are important in diabetes and vitamin D metabolism.

Comparability Subtotal: 1/2 stars

Exposure (max 3 stars)

6. **Ascertainment of exposure**
   ★ 1/1
   Justification*:* Vitamin D status was assessed by measuring serum 25-hydroxyvitamin D (25(OH)D) levels using standard laboratory techniques, providing objective and reliable exposure data.
7. **Same method of ascertainment for cases and controls**
   ★ 1/1
   Justification*:* Both cases and controls underwent the same laboratory procedures for measuring serum 25(OH)D levels, ensuring consistency in exposure assessment.
8. **Non-response rate**
   No star
   Justification*:* The study does not report non-response rates or whether non-respondents were described, leaving this aspect unassessed.

Exposure Subtotal: 2/3 stars

Total = 7/9

4. Adeleye J. et al., 2023

Reference: Adeleye JO, Emuze ME, Esan A, Bamidele OT. Serum vitamin D status and its relationship with insulin resistance amongst a cohort of Nigerian patients with type 2 diabetes mellitus. J Diabetol. 2023;14(4):220–5.

Selection (max 4 stars)

1. **Is the case definition adequate?**
   ★ 1/1
   Justification: Cases were defined as individuals diagnosed with type 2 diabetes mellitus (T2DM) attending the medical outpatient clinic of a hospital in Nigeria. Diagnosis was based on standard clinical criteria, ensuring adequate case definition.
2. **Representativeness of the cases**
   ★ 1/1
   Justification: Participants were selected from a hospital-based medical outpatient clinic, representing a specific patient population. While hospital-based, the selection appears systematic and relevant to the study's objectives.
3. **Selection of controls**
   ★ 1/1
   Justification: Controls were 60 healthy non-diabetic individuals from the same hospital setting, matched by age and sex. This approach minimizes selection bias and provides a suitable comparison group.
4. **Definition of controls**
   ★ 1/1
   Justification: Controls were defined as healthy non-diabetic individuals with no history of diabetes, ensuring they are free from the outcome of interest.

Selection Subtotal: 4/4 stars

Comparability (max 2 stars)

5. **Comparability of cases and controls on the basis of design or analysis**
   ★ 1/2
   Justification: The study matched cases and controls by age and sex. However, it does not report adjustments for other potential confounders such as BMI, physical activity, or dietary factors, which are important in diabetes and vitamin D metabolism.

Comparability Subtotal: 1/2 stars

Exposure (max 3 stars)

6. **Ascertainment of exposure**
   ★ 1/1
   Justification: Vitamin D status was assessed by measuring serum 25-hydroxyvitamin D (25(OH)D) levels using standard laboratory techniques, providing objective and reliable exposure data.
7. **Same method of ascertainment for cases and controls**
   ★ 1/1
   Justification: Both cases and controls underwent the same laboratory procedures for measuring serum 25(OH)D levels, ensuring consistency in exposure assessment.
8. **Non-response rate**
   No star
   Justification: The study does not report non-response rates or whether non-respondents were described, leaving this aspect unassessed.

Exposure Subtotal: 2/3 stars

Total = 7/9

5. Abbiyesuku M. et al., 2016

Reference: Abbiyesuku FM, Olawale OO, Agbakwuru AN, Olooto WE. Vitamin D levels and insulin resistance among Nigerian men with type-2 diabetes mellitus. Ann Heal Res (The J Med Dent Consult Assoc Niger OOUTH, Sagamu, Niger. 2016;2(1):10–6.

Selection (Max 4 stars)

1. **Case definition adequate?**
   ★ 1/1- Cases were adult men with confirmed diagnosis of type 2 diabetes mellitus attending University College Hospital, Ibadan, using standard clinical/biochemical criteria.

2. **Representativeness of the cases?**
   No star - Cases were hospital-based (metabolic research unit) and recruited after consent, but no indication of consecutive or random sampling method; therefore representativeness is uncertain.
3. **Selection of controls?**
   No star -Controls were "apparently healthy men residing in Ibadan and its environment." Community-based, but selection method unspecified (convenience sampling?), so unclear representativeness.
4. **Definition of controls?**
   ★ **1/1**- Controls defined as healthy non-diabetic individuals (no diabetes), satisfying the requirement of no disease (outcome).

Selection subtotal: 2 / 4

Comparability (Max 2 stars)

5. **Comparability of cases and controls (design/analysis)?**
   No star - although "matched for social class" was mentioned, there is no mention of matching or adjustment for age, BMI, or other confounders. In fact, mean age differed significantly between groups (cases older). No multivariable analysis controlling for key factors was reported.

Comparability subtotal: 0 / 2

Exposure (Max 3 stars)

6. **Ascertainment of exposure?**
   ★ **1/1** - Serum 25-OH vitamin D levels measured via laboratory assay - objective exposure measurement.
7. **Same method of ascertainment for cases and controls?**
   ★ **1/1** -Both groups had serum vitamin D measured using the same methods.
8. **Non-response rate?**
   No star -The study does not report participation or non-response rates for either group.

Exposure subtotal: 2 / 3
Total Stars: 4 / 9

6. Abdelsadek, et al., 2018

Reference: Abdelsadek SE, El Saghier EO, Abdel Raheem SI. Serum 25 (OH) vitamin D level and its relation to diabetic peripheral neuropathy in Egyptian patients with type 2 diabetes mellitus. Egypt J Neurol psychiatry Neurosurg. 2018;54(1):36.

Selection (max 4 stars)

1. **Case definition adequate?**
    - ★ **1/1** - Cases are T2DM patients aged 40–60 with clinically confirmed diabetic peripheral neuropathy (DPN) using both clinical assessment and nerve conduction studies (≥ two nerves abnormal)
2. **Representativeness of cases?**
    - ★ **1/1** - Cases were selected from outpatient diabetes and neurology clinics using defined criteria; while hospital-based, the selection method appears systematic from clinic populations
3. **Selection of controls?**
    - ★ **1/1** - Controls were healthy, age- and sex-matched non-diabetic subjects, selected from patients' relatives-reflecting community-like selection rather than clinic-based check-ups
4. **Definition of controls?**
    - ★ **1/1** - Controls were clearly defined as non-diabetic healthy individuals, free from the outcome (DPN)

Selection subtotal: 4 / 4

Comparability (max 2 stars)

5. **Comparability based on design or analysis?**
    - ★ **1/2** - Cases and controls were matched on age and sex, critical confounders. However, the study did not account for other key variables (e.g., BMI, glycemic control, duration of diabetes) in adjustments

Comparability subtotal: 1 / 2

Exposure (max 3 stars)

6. **Ascertainment of exposure?**

    - ★ **1/1** - Serum 25(OH)-vitamin D levels were objectively measured using enzyme immunoassays (EIA)
7. **Same ascertainment method across groups?**
    - ★ **1/1** - The same laboratory methods were applied consistently across cases and controls
8. **Non-response rate?**
    - **No star.** - The study does not report participation or non-response rates for either group.

Exposure subtotal: 2 / 3

Total Score: 7 / 9

7. Batubo U. D et al., 2025

Reference: Batubo UD, Oyan B, Umoren U, Ogbamba S, Abere S, Unachukwu CN. Risk of Insufficient Hydroxyvitamin D Levels in Diabetic Foot Ulcers in Rivers State Nigeria. West Afr J Med. 2025;42(3):225–30.

Selection (max 4 stars)

1. **Is the case definition adequate?**
   ★ 1/1 - Awarded.
   Justification: Cases are clearly defined as patients with diabetic foot ulcers (DFUs) (Wagner grades reported) - a clinical, objective outcome.
2. **Representativeness of the cases**
   No star.
   Justification: The paper reports recruited DFU patients (N=88) but does not state whether cases were consecutive or randomly sampled from the clinic population, so representativeness is uncertain (hospital-based series).
3. **Selection of controls**
   No star.
   Justification: Controls are described as 88 individuals with T2DM without DFUs, but the abstract does not specify whether these controls were community volunteers or hospital clinic patients (selection method unclear). That prevents awarding the community-controls star.
4. **Definition of controls**
   ★ 1/1 - Awarded.

Justification: Controls are explicitly people with T2DM without DFUs (i.e., free of the outcome), satisfying the NOS requirement that controls are defined as not having the outcome.

Selection subtotal: 2 / 4 stars.

Comparability (max 2 stars)

5. **Comparability of cases and controls (design or analysis)**
   No star.
   Justification: The abstract reports regression analysis (an association estimate: OR 12.6, 95% CI 6.0–26.2) but does not specify which covariates (if any) were included in adjusted models or whether groups were matched on key confounders (age, diabetes duration, glycaemic control). Therefore we cannot confirm control for the most important confounder(s).

Comparability subtotal: 0 / 2 stars.

Exposure (max 3 stars)

6. **Ascertainment of exposure**
   ★ 1/1 - Awarded.
   Justification: Serum vitamin D was measured from blood samples "according to standard methods" - objective laboratory exposure assessment.
7. **Same method of ascertainment for cases and controls**
   ★ 1/1 - Awarded.
   Justification: The methods describe measurement of 25(OH)D for both groups using the same laboratory assays, implying consistent ascertainment across cases and controls.
8. **Non-response rate**
   No star.
   Justification: The abstract does not report participation or non-response rates or whether non-respondents were characterized.

Exposure subtotal: 2 / 3 stars.

Total stars: 4 / 9

8. Hassan A. et al., 2024

Reference: Hassan AA, Omar SM, Abdelbagi O, Adam I. Serum 25-Hydroxyvitamin D Concentrations in Patients with Type 2 Diabetes Mellitus in Eastern Sudan: A Case–Control Study. SAGE Open Nurs. 2024;10:23779608241265204.

Selection (max 4 stars)

1. **Is the case definition adequate?**
   ★ 1/1
   Justification: Cases were defined explicitly as *patients with T2DM*. While the abstract doesn't specify the diagnostic criteria (e.g., WHO/ADA fasting glucose), it was conducted in a clinical setting with typical T2DM diagnosis standards implied in published papers of this type. This likely qualifies as adequate.
2. **Representativeness of the cases**
   No star
   Justification: The abstract describes recruitment of 88 cases but does not detail how cases were selected (e.g., randomly or consecutively). Without clarity that cases are representative of the clinic population, selection bias remains possible.
3. **Selection of controls**
   No star
   Justification: Controls are described as *healthy participants matched for age and gender*, but there is no description of their sampling source (community, hospital staff, screening volunteers, etc.). Without clarity, awarding this star is not possible.
4. **Definition of controls**
   ★ 1/1
   Justification: Controls are explicitly healthy (non-diabetic), matched by age/gender, and screened. They clearly represent people *without* the outcome (T2DM).

Selection subtotal: 2 / 4

Comparability (max 2 stars)

5. **Comparability of cases and controls on design or analysis**
   ★ 1 / 2
   Justification: The study matched for age and gender, which addresses key confounding variables. However, the analysis was

univariate only, and other potential confounders (e.g., BMI, education, marital status) were checked and found not different between groups, but no multivariable modeling was performed. Thus only one star awarded.

Comparability subtotal: 1 / 2

Exposure (max 3 stars)

6. **Ascertainment of exposure**
   ★ 1 / 1
   Justification: Serum 25(OH)D levels were measured via laboratory assays, allowing objective measurement of the exposure.
7. **Same method of ascertainment for cases and controls**
   ★ 1 / 1
   Justification: The same laboratory procedures and assays were used on both groups, ensuring consistency.
8. **Non-response rate**
   No star
   Justification: The paper does not provide information on how many individuals were approached or declined participation -non-response rates are not reported.

Exposure subtotal: 2 / 3

Total Score: 5 / 9

9. Arhin-Aidoo F. et al., 2025

Reference: Arhin-Aidoo F, Fondjo LA, Obirikorang C, Owiredu EW, Senu E, Agomuo SKS, et al. Association between vitamin D receptor gene variants and the risk of type 2 diabetes mellitus in a Ghanaian population. Sci Rep. 2025;15(1):26775.

Selection (max 4 stars)

1. **Is the case definition adequate?**
   ★ 1/1

Cases are "clinically diagnosed T2DM patients"-this implies objective clinical/biochemical diagnosis, which meets the standard adequacy requirement.

2. **Representativeness of the cases**
   ★ 1/1
   Cases were recruited using a 1:1 matched design from patients visiting Greenshield Hospital at Sefwi Bekwai. This suggests a defined geographical and clinical base, likely yielding a representative clinical sample.
3. **Selection of controls**
   ★ 1/1
   Controls were "apparently healthy non-diabetic controls living in same environs," indicating community-based selection aligned with the source population.
4. **Definition of controls**
   ★ 1/1
   Controls were healthy and non-diabetic, clearly defined as being free of the outcome (T2DM).

Selection subtotal: 4 / 4

Comparability (max 2 stars)

5. **Comparability of cases and controls (design or analysis)**
   ★ 1/2
   The study employed a 1:1 matching design, likely on age and gender (implied by standard practice, though exact factors matched aren't fully specified). They then applied logistic regression models to assess associations-suggesting some level of control for confounding. However, details on specific covariate adjustment are not provided in the abstract, so only one star is awarded.

Exposure (max 3 stars)

6. **Ascertainment of exposure**
   ★ 1/1
   Serum vitamin D was measured via ELISA, an objective laboratory method.

   Additionally, VDR gene variants were genotyped using PCR-RFLP, another objective exposure measure.

7. **Same exposure assessment for cases and controls**
   ★ 1/1
   The same protocols (ELISA for vitamin D and PCR-RFLP for VDR genotyping) were applied to both groups consistently.
8. **Non-response rate**
   No star
   The abstract does not report on participation rates or non-respondents, so we cannot judge this item.

Exposure subtotal: 2 / 3

Total NOS Score: 7 / 9

10. Errouagui A. et al., 2014

References: Errouagui A, Benrahma H, Charoute H, Ghalim N, Barakat A, Kandil M, et al. Relationship between vitamin d receptor (VDR) gene polymorphisms and susceptibility to Type 2 diabetes mellitus in Moroccans population. Int J Innov Appl Stud. 2014;8(2):503–14.

Selection (Max 4 stars)

1. **Case definition adequate?**
   ★ 1/1 - T2DM clinically diagnosed in patients (reputable standard clinical setting implied).
2. **Representativeness of cases?**
   No star - No details on recruitment (random, consecutive, or otherwise), introducing potential selection bias.
3. **Selection of controls?**
   No star - Healthy controls recruited from community; recruitment method not described clearly to assess representativeness.
4. **Definition of controls?**
   ★ 1/1 - Controls are healthy and non-diabetic (clearly free of outcome).

Selection subtotal: 2 / 4

Comparability (Max 2 stars)

5. **Comparability via design or analysis?**
   ★ **1/2** - Associations estimated, but no mention of adjustment for confounders; matching variables (age, sex) not specified. Therefore, only one star awarded.

Comparability subtotal: 1 / 2

Exposure (Max 3 stars)

6. **Ascertainment of exposure?**
   ★ **1/1** - VDR genotypes PCR-RFLP and serum 25(OH)D via ELISA - objective measures.
7. **Same ascertainment for cases and controls?**
   ★ **1/1** - Methods applied consistently across both groups.
8. **Non-response rate?**
   No star - Not reported in the summary/abstract.

Exposure subtotal: 2 / 3

Total Score: 5 / 9

11. El Gendy H. I. et al., 2018

Referemces: El Gendy HI, Sadik NA, Helmy MY, Rashed LA. Vitamin D receptor gene polymorphisms and 25 (OH) vitamin D: Lack of association to glycemic control and metabolic parameters in type 2 diabetic Egyptian patients. J Clin Transl Endocrinol. 2019;15:25–9.

Selection (Max 4 stars)

1. **Case definition adequate?**
   ★ **1/1** - Cases were patients clinically diagnosed with type 2 diabetes mellitus (registered at Cairo University), implying standard clinical/biochemical diagnostic criteria were used.

2. **Representativeness of the cases**
   No star - The recruiting process (consecutive, random, or otherwise) is not described, so representativeness cannot be confirmed.
3. **Selection of controls**
   No star - Healthy controls were age-matched, but their recruitment method (community/population-based vs convenience sampling) isn't specified.
4. **Definition of controls**
   ★ **1/1** - Controls were healthy non-diabetics, clearly free of the outcome.

Selection subtotal: 2 / 4

Comparability (Max 2 stars)

5. **Comparability of cases and controls**
   ★ **1 / 2** - Groups were age-matched. However, while various metabolic parameters were measured (e.g., BMI, lipids, HbA1c), no multivariable adjustments were reported for confounding effects.

Comparability subtotal: 1 / 2

Exposure (Max 3 stars)

6. **Ascertainment of exposure**
   ★ **1/1** - Genotyping of VDR polymorphisms (FokI, BsmI, TaqI) was done by PCR-RFLP; 25(OH)D levels were measured by ELISA-both objective lab methods.
7. **Same method for cases and controls**
   ★ **1/1** - The same genotyping and ELISA methods applied across both groups.
8. **Non-response rate**
   No star - No information provided about participation or non-response rates.

Exposure subtotal: 2 / 3

Total Score: 5 / 9

Overall case-control studies rating

Study (Author, Year)	Selection (★/Max)/4	Comparability (★/Max)/2	Outcome/Exposure (★/Max)/3	Total (★/Max)/9	Quality Rating*
Mahjoubi I., 2016	2	0	2	4	Low
Melake A., 2025	4	1	2	7	High
Fondjo L., 2017	4	1	2	7	High
Adeleye J., 2023	4	1	2	7	High
Abbiyesuku M., 2016	2	0	2	4	Low
Abdelsadek, 2018	4	1	2	7	High
Batubo U. D., 2025	2	0	2	4	Low
Hassan A., 2024	2	1	2	5	Moderate
Arhin-Aidoo F., 2025	4	1	2	7	High
Errouagui A., 2014	2	1	2	5	Moderate
El Gendy H. I., 2018	2	1	2	5	Moderate

Newcastle–Ottawa Scale (Cross-Sectional Study) – Individual Studies Assessment

1. Aljack, et al. 2019

References: Aljack HA, Abdalla MK, Idris OF, Ismail AM. Vitamin D deficiency increases risk of nephropathy and cardiovascular diseases in Type 2 diabetes mellitus patients. J Res Med Sci. 2019;24(1):47.

Selection (5 stars)

1. **Representativeness of the sample?**
   ★ **1/1** - Patients with T2DM were randomly selected from a military hospital that receives referrals from across Sudan. Likely representative of T2DM population in that setting.
2. **Sample size justification?**
   ★ **1/1** - The study includes a moderate sample size (n = 205), and sample calculation or justification isn't explicitly stated-but size is adequate for statistical analyses they've used, so one star.
3. **Non-selective response rate / response description?**
   ★ **1/1** - Informed consent was obtained; although exact response rate isn't reported, the study states randomly selected and details inclusion/exclusion (e.g., excluding those with inflammatory comorbidities or vitamin D supplementation), suggesting attention to representativeness. I'll award a star.
4. **Ascertainment of exposure (Vitamin D)?**
   ★ **1/1** - Serum 25(OH)D measured via ELISA with sensitivity and protocol details. Objective and valid measure. ([turn1view0])
5. **Ascertainment of outcome (Nephropathy / CVD risk measures)?**
   ★ **1/1** - Outcomes like nephropathy assessed via standardized ACR measurements and hs-CRP; cardiovascular risk inferred through established lab markers-so valid and reliable outcome ascertainment.

Selection total: 5 / 5

Comparability (2 stars)

6. **Control for important confounders?**
   ★ **1/2** - The study assessed potential confounders (e.g., gender, BMI, sunlight exposure, exercise) and performed multiple regression analyses to determine independent associations (e.g., ORs for risk of vitamin D deficiency by BMI, sun exposure). However, it's unclear if analyses adjusted for all key variables in relation to outcomes like nephropathy or CVD risk. So, one star.
7. **Extra adjustment for other factors?**
   No star. - No evidence of controlling for additional confounders like seasonality, sunlight duration by individual, socioeconomic status beyond lifestyle categories.

Comparability total: 1 / 2

Outcome (3 stars)

8. **Assessment of the outcome?**
   ★ **1/1** - Both primary measures ACR and hs-CRP were assessed using established lab instruments (Cobas C-311), ensuring reliability and objectivity.
9. **Statistical test appropriate?**
   ★ **1/1** - Statistical tests included t-tests, chi-square, Pearson's correlation, and multiple regression; appropriate for cross-sectional analyses.
10. **Outcome measured blind or by objective criteria?**
    ★ **1/1** - Outcome measurements (lab-based) were likely blinded to exposure status; methods suggest standard equipment and objective assays. There’s no suggestion of bias during measurement.

Outcome total: 3 / 3

Total Score: 9/10

2. Melake A. et al., 2025

Reference: Melake A, Alamnie G, Mekonnen M. Impact of Vitamin D Deficiency and VDR TaqI Polymorphism on Diabetic Retinopathy Risk Among T2DM Ethiopian Population. Food Sci Nutr. 2025;13(4):e70197.

Selection (max 4 stars)

1. **Is the case definition adequate? - ★ 1/1**
   Justification: Cases were T2DM patients with diabetic retinopathy confirmed by blood-glucose tests and funduscopy, and patients had ≥1 year follow-up at the chronic follow-up clinic.
2. **Representativeness of the cases - ★ 1/1**
   Justification: The authors used an analytical sample-size calculation and simple random sampling from registered clinic patients to recruit cases, which supports representativeness of the clinic population.
3. **Selection of controls - ★ 1/1**
   Justification: Controls were age- and sex-matched healthy non-diabetic volunteers from the same geographical area and social status and were screened (normal blood glucose and funduscopy). This is an appropriate community/healthy control source.
4. **Definition of controls - ★ 1/1**
   Justification: Controls were clearly defined as non-diabetic individuals with normal glucose and normal funduscopy (i.e., free of the outcome).

Selection subtotal: 4 / 4

Comparability (max 2 stars)

5. **Comparability of cases and controls on design/analysis - ★ 1/2**
   Justification: The study matched cases and controls by age and sex, and used logistic regression to examine risk associations. However, the paper does not clearly report multivariable adjusted ORs (or which covariates were included in models), so only one star is awarded for matching/partial control of confounding.

Comparability subtotal: 1 / 2

Exposure (max 3 stars)

6. **Ascertainment of exposure - ★ 1/1**
   Justification: Exposure measures were objective: serum 25(OH)D levels were measured (reported numerically) and DNA was extracted and TaqI genotypes determined by PCR and agarose gel electrophoresis.
7. **Same method of ascertainment for cases and controls - ★ 1/1**
   Justification: The same laboratory/genotyping methods and biochemical tests were applied to both cases and controls.
8. **Non-response rate - No star**
   Justification: Although the sample size calculation allowed for a 10% non-response, the paper does not report how many people were approached vs enrolled or actual non-response rates; therefore this item is unassessable.

Exposure subtotal: 2 / 3

Total Score: 7/10

3. Said J. et al., 2021

Reference: Said J, Lagat D, Kimaina A, Oduor C. Beta cell function, insulin resistance and vitamin D status among type 2 diabetes patients in Western Kenya. Sci Rep. 2021;11(1):4084.

Selection (5 stars)

1. **Representativeness of the sample?**
   ★ **0/1** - Patients were recruited from a clinic population, but no indication they were sampled randomly. Thus, representativeness is uncertain.
2. **Sample size justification?**
   ★ **0/1** - Sample size is moderate (n = 124) but no formal calculation or justification is provided.
3. **Non-response rate / description?**
   ★ **0/1** - The study doesn't report the number of patients approached versus enrolled, so potential response bias isn't assessable.
4. **Ascertainment of exposure (Vitamin D)?**
   ★ **1/1** - Serum 25-OH vitamin D was objectively measured using standard lab assays.

5. **Ascertainment of outcome (HOMA-IR / HOMA-B / DI)?**
   ★ **1/1** - Outcomes were computed using established models (HOMA-IR, HOMA-B, DI) from biochemical measurements, providing objective assessment.

Selection subtotal: 2 / 5

Comparability (2 stars)

6. **Control for important confounders?**
   ★ **1/2** - The study used linear regression adjusting for some confounders (not fully detailed in abstract). Partial adjustment merits one star.
7. **Extra adjustment for additional factors?**
   ★ **0/2** - No indication of adjustment for other potential confounders (e.g., BMI, duration of T2DM, season), so no second star.

Comparability subtotal: 1 / 2

Outcome (3 stars)

8. **Assessment of the outcome?**
   ★ **1/1** - Measurements (fasting glucose, insulin) likely standardized and reliable, forming the basis for HOMA models.
9. **Statistical test appropriate?**
   ★ **1/1** - The use of linear regression and correlation is appropriate for evaluating continuous relationships.
10. **Outcome measured with objective criteria or blinded measurement?**
    ★ **1/1** - Laboratory-derived and model-based outcomes reduce measurement bias. No subjective measurement involved.

Outcome subtotal: 3 / 3

Total Score: 6/10

4. Karau, et al., 2019

Reference: Karau PB, Kirna B, Amayo E, Joshi M, Ngare S, Muriira G. The prevalence of vitamin D deficiency among patients with type 2 diabetes seen at a referral hospital in Kenya. Pan Afr Med J. 2019;34.

Selection (5 points)

1. **Representativeness - ★ 1/1**
   → Patients were on follow-up at a tertiary referral hospital, likely covering a broad clinical population of T2DM patients in Nairobi.
2. **Sample size justification - ★ 0/1**
   → Study did not provide a formal sample size calculation.
3. **Non-response rate - ★ 0/1**
   → No data on participation rate or numbers approached vs enrolled.
4. **Ascertainment of exposure (Vitamin D measurement) - ★ 1/1**
   → Serum 25(OH)D measured objectively via blood test.
5. **Ascertainment of outcome (glycaemic control, BMI) - ★ 1/1**
   → HbA1c, BMI, and waist–hip ratio measured using standardized methods.

Selection subtotal: 3 / 5

Comparability (2 points)

6. **Control for important confounders - ★ 1/2**
   → Study explored correlations and found associations but did not perform multivariable analysis to adjust for confounders; partial merit for analysis.
7. **Adjustment for additional factors - ★ 0/2**
   → No mention or control for other potential confounders (e.g., seasonality, diet, sunlight exposure, socioeconomic status).

Comparability subtotal: 1 / 2

Outcome (3 points)

8. **Outcome assessment call - ★ 1/1**
   → Clinical lab measures (HbA1c, BMI, etc.) were recorded objectively.
9. **Statistical test appropriateness - ★ 1/1**
   → Pearson correlation used for continuous variables; appropriate for cross-sectional study.
10. **Objective measurement/blinded assessment - ★ 1/1**
   → Lab-based, analytical measures reduce measurement bias.

Outcome subtotal: 3 / 3

Total Score: 7/10

5. Fondjo L. et al., 2018

Reference: Fondjo LA, Sakyi SA, Owiredu WKBA, Laing EF, Owiredu EW, Awusi EK, et al. Evaluating vitamin D status in pre-and postmenopausal type 2 diabetics and its association with glucose homeostasis. Biomed Res Int. 2018;2018(1):9369282.

Selection (5 points)

1. **Representativeness of the sample?**
   ★ 1/1
   Justification: The study used Fischer’s formula to calculate sample size and recruited 192 consecutively selected T2DM women (both pre- and postmenopausal) attending a large teaching hospital clinic, which suggests representativeness of the clinical population
2. **Sample size justification?**
   ★ 1/1
   Justification: The sample size was calculated using a standard statistical method (Fischer’s formula), demonstrating methodological rigor
3. **Non-selection/response rate?**
   ★ 0/1
   Justification: There is no information on how many patients were approached versus enrolled or non-participation, so response bias cannot be assessed.

4. **Ascertainment of exposure (vitamin D measurement)?**
   ★ 1/1
   Justification: Serum 25(OH)D was measured using ELISA, a validated and objective laboratory method
5. **Ascertainment of outcome (glycemic control measures)?**
   ★ 1/1
   Justification: Outcomes included FBG, insulin, HbA1c, lipid profile, and calcium-all measured using validated laboratory techniques

Selection subtotal: 4 / 5

Comparability (2 points)

6. **Control for key confounders?**
   ★ 1/2
   Justification: The study assessed and reported associations stratified by menopausal status and performed regression analyses to identify risk factors of hypovitaminosis D (e.g., BMI, duration of diabetes, waist-to-height ratio). However, not all potential confounders (e.g., season, dietary intake, physical activity) were adjusted in the glycemic outcome models
7. **Additional adjustments?**
   ★ 0/2
   Justification: No evidence of adjustment for other important lifestyle or environmental confounders beyond the metabolic and demographic factors measured.

Comparability subtotal: 1 / 2

Outcome (3 points)

8. **Assessment of outcomes?**
   ★ 1/1
   Justification: Outcomes were measured objectively using standard clinical assays (e.g., ELISA for insulin, FBG analyzer)
9. **Statistical tests appropriate?**
   ★ 1/1

Justification: They used regression analyses, correlation, and appropriate tests for continuous data (R^2, p-values) to assess associations - statistically sound for cross-sectional data

10. **Objective/blinded outcome measurement?**
    ★ 1/1
    Justification: Laboratory measurements were objective. While blinding isn't explicitly stated, lab-based assays minimize bias.

Outcome sub/total: 3 / 3

Total Score: 8/10

6. Raharinavalona A. et al., 2024

Reference: Raharinavalona SA, Raherison RE, Miandrisoa RM, Andrianasolo RL, Rakotomalala ADP. Vitamin D Status and Cardiovascular Risk Factors in Patients with Type 2 Diabetes Mellitus: A Cross-Sectional Study in a Tertiary-Level Hospital in Antananarivo, Madagascar. Diabetes, Metab Syndr Obes. 2024;2191–8.

Selection (5 stars)

1. **Representativeness of the sample - ★ 1/1**
   Justification: 318 consecutive patients with T2DM attending a large tertiary hospital-likely representative of the clinic population. (Implicit in recruitment context.)
2. **Sample size justification - ★ 0/1**
   Justification: The abstract does not indicate a formal sample size calculation.
3. **Non-response rate / description - ★ 0/1**
   Justification: No data on how many were approached versus enrolled or refusal rates.
4. **Exposure ascertainment (Vitamin D measurement) - ★ 1/1**
   Justification: Vitamin D was measured using chemiluminescence-an objective, valid lab method. ([turn0search0])
5. **Outcome ascertainment (CRF and complications) - ★ 1/1**
   Justification: Outcomes (hypertension, dyslipidemia, microalbuminuria, etc.) were apparently assessed via objective clinical/lab methods, though full detail not in abstract-still acceptable.

Selection subtotal: 3 / 5

Comparability (2 stars)

6. **Control for crucial confounders - ★ 1/2**
   Justification: The study adjusted associations for age, HbA1c, and retinopathy in multivariable analysis, controlling for key confounders.
7. **Control for additional confounders - ★ 0/2**
   Justification: No indication that further lifestyle or socioeconomic variables were adjusted.

Comparability subtotal: 1 / 2

Outcome (3 stars)

8. **Assessment of outcome - ★ 1/1**
   Justification: Outcomes such as hypertension, dyslipidemia, etc., are typically assessed via standardized clinical definitions- appropriate for cross-sectional studies.
9. **Statistical test appropriateness - ★ 1/1**
   Justification: Use of multivariable logistic regression is suitable and appropriate.
10. **Objective/blinded outcome measurement - ★ 1/1**
    Justification: Lab and clinical measurements reduce measurement bias; though blinding is not stated, the assessments are objective.

Outcome subtotal: 3 / 3

Total Score: 7/10

7. Erasmus R. et al., 2022

Reference: Erasmus R, Maepa S, Machingura I, Davids S, Raghubeer S, Matsha T. Vitamin D, vitamin D-binding proteins, and VDR polymorphisms in individuals with hyperglycaemia. Nutrients. 2022;14(15):3147.

Selection (5 stars)

1. **Representativeness of the sample? ★ 1/1**
   → Large community-based sample (n=968) from a well-defined urban population, with exclusions clearly stated.
2. **Sample size justification? ★ 1/1**
   → Sample derived from larger Vascular and Metabolic Health cohort; participant number is substantial and methodologically justified through cohort design.
3. **Non-response rate / description? ★ 0/1**
   → No explicit reporting of response rates or numbers approached vs accepted.
4. **Ascertainment of exposure (Vitamin D measurement)? ★ 1/1**
   → Serum 25(OH)D measured objectively using chemiluminescence assays; high validity.
5. **Ascertainment of outcome (glycaemic status, VDR SNPs)? ★ 1/1**
   → Glycaemic status determined by OGTT; VDR genotypes validated in independent lab. Robust outcome assessment.

Selection subtotal: 4 / 5

Comparability (2 stars)

6. **Control for key confounders? ★ 1/2**
   → Utilized multiple linear regression modeling to evaluate associations, but it's unclear whether lifestyle or demographic confounders were fully adjusted.
7. **Additional factor adjustment? ★ 0/2**
   → No reporting of adjustment for other important confounders like seasonality, physical activity, or socioeconomic status.

Comparability subtotal: 1 / 2

Outcome (3 stars)

8. **Assessment of outcomes? ★ 1/1**
   → Objective measurements for vitamin D, binding proteins, and validated genotyping methods ensure reliable outcomes.

9. **Appropriate statistical tests? ★ 1/1**
   → Used chi-square tests for genotype/disease associations and multiple linear regression for continuous variables-appropriate for design.
10. **Outcome measured objectively? ★ 1/1**
   → All lab-based measurements and standardized assays; minimal bias risk.

Outcome subtotal: 3 / 3

Total Score: 8/10

8. Mostafa, et al., 2023

Reference: Mostafa NR, Ali AAM, Marzo RR. The effect of vitamin D deficiency on glycemic control in patients with type 2 diabetes mellitus. Healthc Low-resource Settings. 2023;11(s2).

Selection (5 points)

1. **Representativeness of sample?**
   ★ 1/1 - Patients were randomly selected from the outpatient clinic or inpatient wards. Random selection from the target T2DM population reduces selection bias.
2. **Sample size justification?**
   ★ 0/1 - No formal sample size calculation is mentioned.
3. **Non-response rate / description?**
   ★ 0/1 - No information on refusal or number of patients approached vs enrolled.
4. **Exposure ascertainment (Vitamin D measurement)?**
   ★ 1/1 - Vitamin D assessed objectively using HPLC, a gold-standard method.
5. **Outcome ascertainment (glycemic control, labs)?**
   ★ 1/1 - FBS and HbA1c measured via validated laboratory techniques; other labs included standard blood chemistry.

Selection subtotal: 3 / 5

Comparability (2 points)

6. **Control for important confounders?**
   ★ **0/2** - Study compares vitamin D levels with FBS/HbA1c but does not adjust for potential confounders (age, sex, disease duration, diet, physical activity).
7. **Additional factor adjustment?**
   ★ **0/2** - No additional adjustments reported.

Comparability subtotal: 0 / 2

Outcome (3 points)

8. **Assessment of outcomes?**
   ★ **1/1** - Outcomes measured objectively using lab assays (HPLC for vitamin D, standard FBS/HbA1c tests).
9. **Statistical test appropriateness?**
   ★ **1/1** - Paired t-tests and Wilcoxon signed-rank tests appropriate for comparison between groups or time periods.
10. **Objective/blinded measurement?**
   ★ **1/1** - Lab-based measures minimize bias; blinding not explicitly stated but objective assays are sufficient.

Outcome subtotal: 3 / 3

Total Score: 6/10

9. Anyanwu A. et al., 2020

Reference: Anyanwu AC, Olopade OB, Onung SI, Odeniyi IA, Coker HAB, Fasanmade OA, et al. Serum vitamin D levels in persons with type 2 diabetes mellitus in Lagos, Nigeria. 2020;

Selection (5 points)

1. **Representativeness of the sample:**
   Score: 1/1
   The study included patients from a specialized diabetes clinic, providing a relevant sample of T2DM patients in Lagos.
2. **Sample size justification:**
   Score: 0/1
   The study does not mention a power calculation or rationale for the sample size.
3. **Non-response rate:**
   Score: 0/1
   No information is provided regarding non-response or refusal rates.
4. **Ascertainment of exposure (Vitamin D measurement):**
   Score: 1/1
   Serum vitamin D levels were measured using HPLC, a reliable and objective method.
5. **Ascertainment of outcome (glycemic control):**
   Score: 1/1
   HbA1c and fasting plasma glucose levels were measured using standard laboratory techniques.

Total for Selection: 3/5

Comparability (2 points)

6. **Control for confounding factors:**
   Score: 0/2
   The study does not report adjusting for potential confounders such as age, sex, BMI, or duration of diabetes.

Total for Comparability: 0/2

Outcome (3 points)

7. **Assessment of outcomes:**
   Score: 1/1
   Outcomes were measured using validated laboratory methods.

8. **Statistical test appropriateness:**
   Score: 1/1
   The statistical tests used (t-tests, Z-tests) are appropriate for comparing means and proportions.
9. **Objective measurement:**
   Score: 1/1
   Laboratory measurements are objective, and the study does not mention blinding, which is acceptable for this type of analysis.

Total for Outcome: 3/3

Total Score: 6/10

10. Safi S. et al., 2015

Reference: Safi S, Ouleghzal H, Khaldouni I, Hassikou H, Ballouch L, Bamou Y, et al. Statut de la vitamine D chez les patients diabétiques de type 2 marocains. Médecine des Mal Métaboliques. 2015;9(1):67–72.

Selection (5 points)

1. **Representativeness of the sample:** ★ 1/1
   - Patients recruited from a specialized hospital; relevant sample of T2DM population.
2. **Sample size justification:** ★ 0/1
   - No formal sample size calculation reported.
3. **Non-response rate:** ★ 0/1
   - No information on refusals or non-response.
4. **Exposure ascertainment (Vitamin D measurement):** ★ 1/1
   - Serum 25(OH)D measured via HPLC, reliable and objective.
5. **Outcome ascertainment (glycemic control):** ★ 0/1
   - Glycemic control measurements not clearly detailed.

Selection subtotal: 2/5

Comparability (2 points)

6. **Control for confounders:** ★ 0/2
    - No adjustment for age, sex, BMI, or diabetes duration.

Comparability subtotal: 0/2

Outcome (3 points)

7. **Assessment of outcomes:** ★ 1/1
    - Lab measurements of vitamin D are valid.
8. **Statistical test appropriateness:** ★ 1/1
    - t-tests and Z-tests appropriate for group comparisons.
9. **Objective measurement:** ★ 1/1
    - Lab-based objective measures; blinding not mentioned but acceptable.

Outcome subtotal: 3/3

Total Score: 5/10

11. Hasan A. et al., 2022

Reference: https://bucket.theses-algerie.com/files/repositories-dz/a471dd00-b901-4f2a-a913-fcc600e3693f.pdf

Selection (5 points)

1. **Representativeness of the sample:** The study includes 100 T2DM patients from Mostaganem City, providing a relevant sample for the Algerian population.
   Score: 1/1
2. **Sample size justification:** The thesis does not provide a power calculation or rationale for the chosen sample size.
   Score: 0/1

3. **Non-response rate:** No information is provided regarding non-responses or refusals.
   Score: 0/1
4. **Ascertainment of exposure (vitamin D levels):** Serum 25(OH)D levels were measured using standardized laboratory methods.
   Score: 1/1
5. **Ascertainment of outcome (HbA1c levels):** HbA1c levels were measured using standardized laboratory methods.
   Score: 1/1

Total for Selection: 3/5

Comparability (2 points)

6. **Control for confounders:** The study does not adjust for potential confounders such as age, sex, BMI, or duration of diabetes.
   Score: 0/2

Total for Comparability: 0/2

Outcome (3 points)

7. **Assessment of outcomes:** HbA1c and 25(OH)D levels were measured using reliable and validated laboratory methods.
   Score: 1/1
8. **Statistical test appropriateness:** The study uses appropriate statistical tests to analyze the data.
   Score: 1/1
9. **Non-respondents:** The study does not report on non-respondents or losses to follow-up.
   Score: 0/1

Total for Outcome: 2/3

Total Score: 5/10

Overall cross-sectional studies rating

**Study (Author, Year)**	**Selection (★/Max) /5**	**Comparability (★/Max)/2**	**Outcome/Exposure (★/Max)/3**	**Total (★/Max) /10**	**Quality Rating***
Aljack, 2019	5	1	3	9	High
Melake A., 2025	4	1	2	7	High
Said J., 2021	2	1	3	6	Moderate
Karau, 2019	3	1	3	7	High
Fondjo L., 2018	4	1	3	8	High
Raharinavalona A., 2024	3	1	3	7	High
Erasmus R., 2022	4	1	3	8	High
Mostafa, 2023	3	0	3	6	Moderate
Anyanwu A., 2020	3	0	3	6	Moderate
Safi S., 2015	2	0	3	5	Moderate
Hasan A., 2022	3	0	2	5	Moderate

Summary of NOS Appraisal

Quality Category	Number of Studies	Percentage (%)
High (≥7 stars)	11	50
Moderate (5–6)	8	36.4
Low (≤4 stars)	3	13.6
