# Supplementary figures and images for: Hidden deficiency under bright skies: Vitamin D prevalence and genetic associations in African Type 2 diabetes: A systematic review and meta-analysis
Source: PLoS One. 2026 Jul 24;21(7):e0354518. doi: 10.1371/journal.pone.0354518 (PMC13399315; doi:10.1371/journal.pone.0354518)

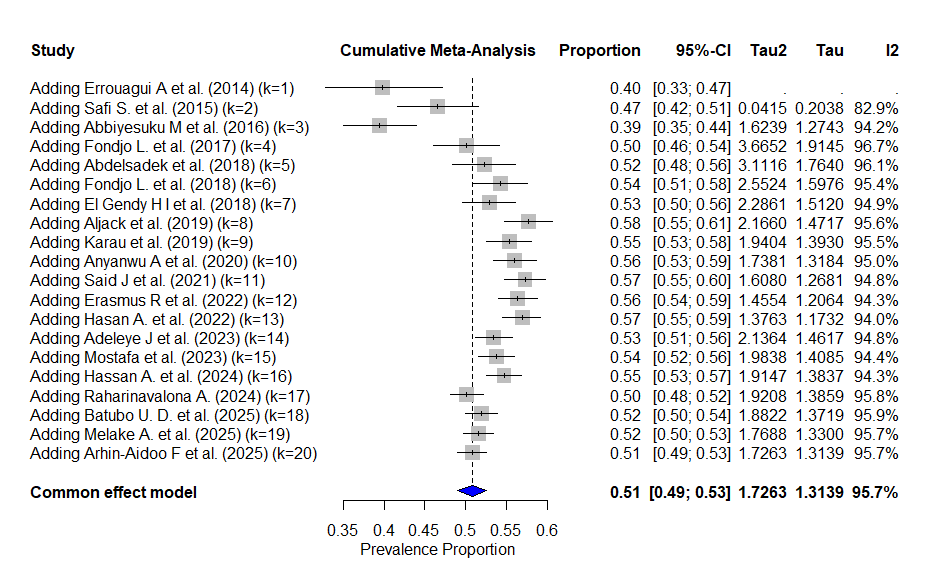

Supplement: S1 Fig — (TIF) [file pone.0354518.s007.tif]

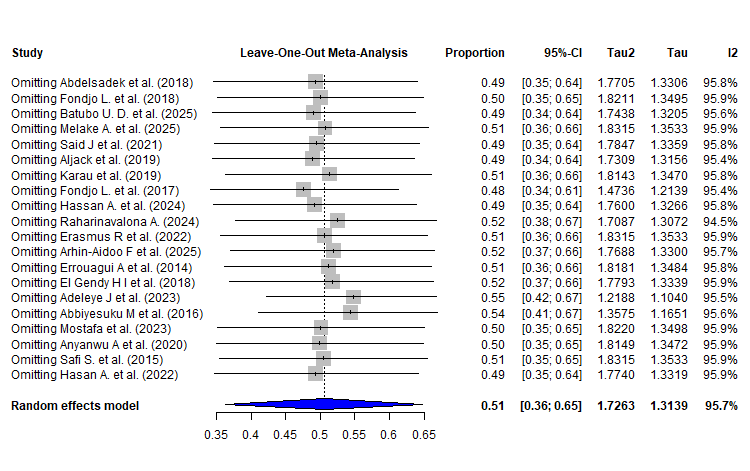

Supplement: S2 Fig — (TIF) [file pone.0354518.s008.tif]

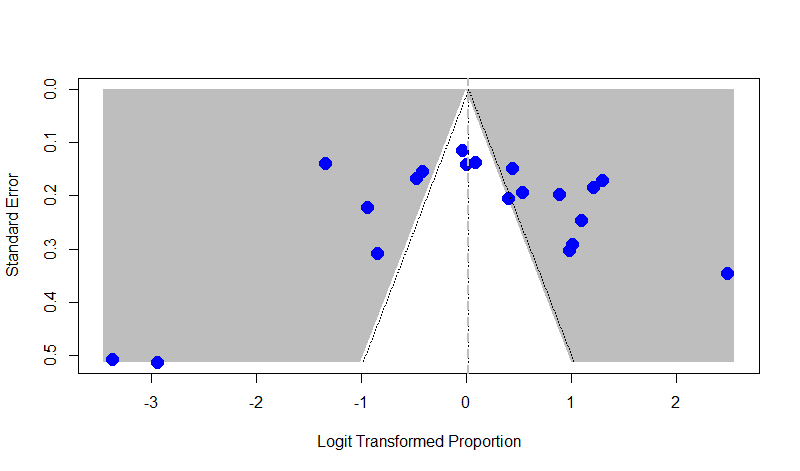

Supplement: S3 Fig — (TIF) [file pone.0354518.s009.tif]
