# Supplementary material for: Hidden deficiency under bright skies: Vitamin D prevalence and genetic associations in African Type 2 diabetes: A systematic review and meta-analysis
Source: PLoS One. 2026 Jul 24;21(7):e0354518. doi: 10.1371/journal.pone.0354518 (PMC13399315; doi:10.1371/journal.pone.0354518)
Supplement: Attachment — Submitted filename: Response to Reviewers.pdf [file pone.0354518.s012.pdf]

Point-by-point response

Dear Editor-in-Chief,

Thank you for coordinating the review of our manuscript. We sincerely appreciate the reviewers' insightful and constructive comments. We have carefully addressed all feedback and have prepared a point-by-point response, with revisions highlighted in green within the manuscript. The manuscript has been updated accordingly, to reflect these changes.

Journal Requirements

1. Please ensure that your manuscript meets PLOS ONE's style requirements, including those for file naming.

   Response: Thank you for the comment. We have updated the manuscript accordingly to comply with the journal formatting requirements, including the file naming.

2. Thank you for stating the following in the Competing Interests section: The authors report no conflicts of interest in this work.

   Please confirm that this does not alter your adherence to all PLOS ONE policies on sharing data and materials, by including the following statement: "This does not alter our adherence to PLOS ONE policies on sharing data and materials."

   Please include your updated Competing Interests statement in your cover letter; we will change the online submission form on your behalf.

   Response: Thank you for the clarification. We have updated the Competing Interests section accordingly in both the manuscript and the cover letter.

3. If the reviewer comments include a recommendation to cite specific previously published works, please review and evaluate these publications to determine whether they are relevant and should be cited. There is no requirement to cite these works unless the editor has indicated otherwise.

   Response*:* We note the editor's suggestion regarding the citation of previously published works. In line with Reviewer 3's feedback (comment 6), we have included citations for the GRADE framework in the Methods section to provide readers with guidance on the tool used to assess certainty of evidence. No other additional citations were added.

Reviewer #1:

1. This manuscript presents a well-designed and timely systematic review and meta-analysis addressing vitamin D deficiency, glycemic control, and VDR polymorphisms among individuals with type 2 diabetes in African populations. The study follows PRISMA 2020 guidelines, has a registered PROSPERO protocol, applies appropriate statistical methods, and addresses an important gap in the literature. Overall, the manuscript is methodologically sound and largely suitable for publication in PLOS ONE, pending minor but targeted revisions aimed at improving clarity and interpretation.

   Response*: We thank the reviewer for the positive and constructive feedback. We have addressed the suggested revisions to improve clarity and interpretation as indicated.*

2. Definition and standardization of vitamin D deficiency

   Vitamin D deficiency is a primary outcome of this review; however, the included studies apply different cut-off values to define deficiency (e.g., <20 ng/mL vs. <30 ng/mL). The manuscript would benefit from a clearer explanation of how this variability was handled analytically. If feasible, a sensitivity or subgroup analysis based on vitamin D cut-off definitions could be considered. Alternatively, this issue should be more explicitly acknowledged as a contributor to between-study heterogeneity in the Limitations section.

   Response*: Thank you for your comment. We have now addressed the variability in vitamin D deficiency cutoffs by adding the distribution of cutoff definitions in the Study characteristics section, conducting sensitivity analyses showing no significant association between cutoffs and study characteristics, and acknowledging this variation as a source of clinical heterogeneity in the Limitations section.*

3. Interpretation of substantial heterogeneity

   The pooled prevalence analysis demonstrates very high heterogeneity (I^2 >95%). While meta-regression and subgroup analyses were conducted, the Discussion could be strengthened by a more explicit interpretation of the implications of this heterogeneity. In particular, the authors may wish to clarify how such variability affects the generalizability of the findings and their relevance for regional or clinical decision-making in African contexts.

   Response*: We thank the reviewer for the comment. We have included a statement in the Discussion section under Limitations addressing the high heterogeneity and its implications for generalizability and clinical relevance in African populations.*

4. Scope and interpretation of genetic findings

   The conclusions regarding the lack of association between VDR FokI polymorphism and type 2 diabetes risk are appropriate; however, they would benefit from additional contextualization. Given the small number of genetic studies and the extensive genetic diversity across African populations, the authors are encouraged to explicitly frame these findings as hypothesis-generating and to emphasize the need for larger, population-specific genetic studies.

   Response: *Thank you for the suggestion. We have revised the Conclusion to frame the findings on VDR FokI polymorphism as hypothesis-generating and emphasize the need for larger, population-specific genetic studies across Africa.*

5. Data availability and reproducibility

   The Data Availability Statement is acceptable; however, the authors are encouraged to clarify whether extracted datasets and/or statistical code (e.g., R scripts) could be made available as supplementary material. This would further enhance transparency and reproducibility, in line with PLOS ONE's open science principles.

   Response: *Thank you for the suggestion. We have included a supplementary file containing both the extracted dataset and the complete R script used for all analyses reported in the manuscript.*

Reviewer #2:

1. This systematic review addresses an important and understudied topic — vitamin D deficiency (VDD) in African populations with type 2 diabetes mellitus (T2DM). The authors followed PRISMA and JBI guidelines, pre-registered the protocol, and performed a range of appropriate analyses. However, several major analytical concerns substantially limit the interpretability of the findings and require revision before acceptance.

   ***Response**: We thank the reviewer for recognizing the importance of this topic and for acknowledging the methodological rigor of our work. We appreciate the constructive feedback and address the analytical concerns raised to improve the clarity and interpretability of our findings.*

2. VDD Definition Not Clearly Specified: The paper never clearly states how VDD was defined across studies or whether a unified threshold was applied for pooling. This is a critical methodological detail that must be reported transparently in the methods section or a supplementary table.

   ***Response**: Thank you for your comment. We have addressed the VDD definition by: (1) adding the distribution of cutoff definitions in the Study characteristics section, (2) including each study's specific cutoff in Table 1, (3) conducting sensitivity analyses showing no significant association between cutoffs and study characteristics, and (4) acknowledging this variation as a source of clinical heterogeneity in the Limitations section.*

3. Case-Control Studies and Prevalence Estimation: Eleven of the 22 included studies used a case-control design, from which prevalence estimates should not be directly calculated, as prevalence in such designs is a function of the sampling ratio rather than true population prevalence. It is unclear whether VDD prevalence estimates were drawn only from the "case" (T2DM) arm of these studies and, if so, how they were handled differently from cross-sectional estimates. The subgroup analysis by study design is appreciated but does not resolve this concern, which should be explicitly addressed in the methods.

   ***Response**: We thank the reviewer for this important methodological observation. For case–control studies, prevalence estimates were calculated using only the T2DM ('case') group to ensure that the estimates reflect the target population. For example, Abdelsadek 2018 included 60 T2DM patients and 20 controls, with 73.3 % of cases being vitamin D deficient. Similarly, Fondjo 2017 reported 92.4 % deficiency among 118 T2DM cases versus 60.2 %*

in controls; only the cases were included in our pooled prevalence analysis. The revised Methods section now explicitly describes how prevalence estimates from case–control studies were handled.

4. Inclusion of a Non-Peer-Reviewed Source: Reference 34 (Hasan, Algeria) appears to be a thesis rather than a peer-reviewed publication. Its inclusion should be explicitly justified, and its methodological quality should be assessed transparently, as these are not subject to the same review process as journal articles.

   ***Response**: Thank you for this observation. Reference 34 (Hasan, Algeria) is a thesis included to provide geographical representation of VDD prevalence in Algeria. Its methodological quality was assessed using the same Newcastle–Ottawa Scale applied to all included studies, and it received the lowest score (5 stars) compared with the other cross-sectional studies, which is now noted in the Methods section for transparency.*

5. Extreme Heterogeneity: The pooled prevalence of 51% (95% CI: 36–65%) is accompanied by $I^2 = 95.7\%$, which is extreme. This is not merely a limitation to acknowledge — it fundamentally undermines the validity of a single pooled estimate. When I^2 is this high, the confidence interval is almost entirely driven by between-study variance (τ^2), and the pooled figure of 51% carries very little real estimative value. While the authors acknowledged heterogeneity, conducted subgroup analyses by region and study design, and ran a meta-regression on publication year and sample size, none of these explained the variability. The authors should have explored the most clinically plausible sources of heterogeneity as moderators in the meta-regression — specifically: VDD threshold definition (e.g., <20 ng/mL vs. <30 ng/mL vs. <50 nmol/L), assay method (immunoassay vs. LC-MS/MS), season of blood draw, and diabetes duration. These were mentioned in the discussion as speculative explanations but were never formally tested, which represents a significant analytical gap. Furthermore, in case there is insufficient data to assess confounders, the authors should explicitly discuss whether pooling these studies is even appropriate at this level of heterogeneity, or whether a narrative synthesis would be more honest for certain outcomes.

   ***Response**: We thank the reviewer for highlighting the issue of extreme heterogeneity. We acknowledge that the pooled prevalence estimate ($I^2 = 95.7\%$) reflects substantial between-study variability. In addition to the preexisting subgroup analyses, we conducted formal subgroup analyses and meta-regression by vitamin D cutoff definition. While the full subgroup analysis showed significant differences ($p = 0.002$), a binary comparison of <20*

ng/mL versus ≥20 ng/mL revealed no significant difference (p = 0.373), and meta-regression treating the cutoff as a continuous variable confirmed it did not explain heterogeneity (p = 0.473). These analyses are now included in the revised manuscript.

Other clinically plausible moderators suggested by the reviewer (assay method, season of blood draw, and diabetes duration) were insufficiently or inconsistently reported across studies and therefore could not be formally tested. We have clarified this limitation in the manuscript.

We agree that the high heterogeneity limits the interpretability of a single pooled estimate; however, synthesizing the available evidence remains valuable for highlighting the substantial variability in VDD prevalence across African T2DM populations and for identifying important methodological gaps that future studies should address.

6. VDR FokI Polymorphism Analysis: Only 4 studies were pooled for the VDR FokI analysis, yielding I^2 = 80.8% and a confidence interval so wide, making it analytically not meaningful: OR = 1.51 (95% CI: 0.23–9.73). While the GRADE rating of "very low" certainty is appropriate, the language used in the abstract and conclusion do not adequately reflect this uncertainty. For instance, lines 45–46 conclude that "current evidence does not support a clear influence of VDR FokI polymorphisms on T2DM susceptibility". This statement that is too confident given the extreme imprecision. A more accurate conclusion would be that the available evidence is insufficient to draw any meaningful conclusion on this outcome.

   ***Response**: Thank you for this observation. While the original wording suggested a lack of clear evidence, we agree that it may be interpreted as implying absence of effect. We have therefore revised the Abstract and Conclusion to more clearly reflect the uncertainty, stating that the current evidence is insufficient to draw meaningful conclusions regarding the association between VDR FokI polymorphisms and T2DM susceptibility.*

7. HbA1c Association: The finding that VDD is associated with higher HbA1c (MD = 0.89%, 95% CI: 0.06–1.72, I^2 = 0%) is based on only 3 studies. Although the I^2 = 0% is reassuring, three studies are far too few to draw reliable conclusions, and this result should be treated as preliminary and hypothesis-generating. The confidence interval nearly crosses zero, indicating meaningful imprecision. Despite the appropriate GRADE rating of "low" certainty, the language in the abstract and conclusions present this finding with more confidence than is warranted (lines 291–292: "significantly associated with poorer

glycemic control"; line 317: "The significant association between VDD and poorer glycemic control"). This language should be softened accordingly. Additionally, it is unclear whether the three included studies adjusted for key confounders such as diabetes duration, medication use, BMI, or renal impairment — all of which affect vitamin D levels and glycemic control. Unadjusted mean differences in observational studies are highly susceptible to confounding, and this issue is not discussed.

***Response**: Thank you for this important comment. We have revised the Discussion to better reflect the uncertainty of this finding. Specifically, we clarified that the HbA1c association is based on a small number of studies and should be interpreted with caution. We also acknowledged the lack of consistent adjustment for key confounders (such as diabetes duration, medication use, BMI, and renal function), and emphasized that this result should be considered hypothesis-generating rather than conclusive.*

8. Meta-Regression: The meta-regression included only publication year and sample size as covariates across 20 studies. While borderline acceptable in terms of the study-to-covariate ratio, the two covariates chosen are arguably the least likely to explain heterogeneity in VDD prevalence. The failure to include more clinically meaningful predictors such as VDD threshold used, assay type, or study setting as moderators (if data were available in the extracted tables) is a missed opportunity and substantially weakens this section.

***Response:** We thank the reviewer for this insightful comment. In addition to the original meta-regression on publication year and sample size, we conducted meta-regression using vitamin D cutoff thresholds as a continuous variable. This analysis showed that variation in cutoff values did not significantly explain heterogeneity ($p = 0.473$). We acknowledge that other clinically relevant moderators, such as assay type or study setting, could not be formally assessed due to limited reporting in the primary studies. We have updated the Methods and Results sections to describe this additional analysis and explicitly note these limitations.*

9. Line 311: The claim that African nations "contributed only five studies" to a prior global review, despite 10 eligible studies existing before that date, should be supported with a supplementary comparison table that also addresses differences in inclusion/exclusion criteria between the two reviews.

***Response**: We thank the reviewer for this suggestion. Instead of creating a separate table, we have included the relevant information on African studies from the prior global review*

directly in our supplementary extracted studies table (S5 File). This approach allows readers to easily compare study coverage between reviews.

10. References: Line 409: Reference 1 ("Analytical Fact Sheet…") is incomplete — no author, year, or URL is provided. This must be corrected.

    Response*: We thank the reviewer for noticing this. The reference has been updated accordingly*

11. Line 120: The exclusion criterion referencing "sample sizes too small to generate meaningful estimates" does not specify the cutoff used. This threshold should be defined.

    Response*: We appreciate this observation and have clarified the exclusion criterion by specifying that studies with fewer than 50 participants were considered too small to generate meaningful estimates.*

12. Seasonal variation as a potential confounding factor in vitamin D levels is not formally addressed and should be discussed or tested where data permit.

    Response*: Thank you for this suggestion. We have updated the discussion section accordingly.*

13. Line 323: A period is missing at the end of the sentence.

    Response*: We thank the reviewer for noticing this. The sentence has been updated accordingly.*

Reviewer #3:

1. This a review of "Hidden deficiency under bright skies: Vitamin D prevalence and genetic associations in African type 2 Diabetes: A systematic review and meta-analysis." This study is a review of the current literature that explores the link between the presence of type 2 diabetes mellitus (T2DM) and vitamin D deficiency (VDD) within several African nations. The author's identified 22 appropriate studies of with 3,447 total individuals from 11 African nations as being relevant to their research question. Their analysis of the relevant papers showed a link between the presence of VDD and T2DM and recommend that reducing VDD could be an effective compliment to diabetes management in Africa. The study is well done and the authors did a thorough job of identifying and screening for relevant studies and in the subsequent analyses of said studies. I have a few general comments and some remarks regarding the writing, all of which are minor.

   ***Response**: We appreciate the reviewer's positive feedback and have incorporated the comments accordingly.*

2. Line 98: Add spaces between 1 & 2

   ***Response**: Thank you, we have revised accordingly.*

3. Line 108-109: The "as well as we have" in this sentence sounds weird consider changing it to "and we have" or something similar

   ***Response**: Thank you, we have revised accordingly.*

4. Line 132-133: Is this PRISMA flow diagram the same as what is shown in Figure 1? Could site Figure 1 here

   ***Response**: Thank you for the suggestion. We have now cited Figure 1 in the Methods section to improve clarity, while retaining the full figure title in the Results section in accordance with the journal's formatting guidelines.*

5. Line 175: change to "explore the source"

   ***Response**: Thank you, we have revised accordingly.*

6. Line 177-182: I was not familiar with the GRADE framework that was mentioned in this section and had to look it up. I think this section could use a citation or two about the framework and a little more expansion on how the up/downgrading is established to acquaint the reader with the tool. This extends to the Certainty of Evidence section starting on Line 275. Does the GRADE framework offer a value to place it in the moderate category? Likewise are we assuming that everything starts at the highest category hence

your language of "downgraded for substantial heterogeneity". Based on that it seems that way.

***Response**: Thank you for the comment. We have added citations for the GRADE framework (Hultcrantz et al., 2017; Schünemann et al., 2020) and clarified in the Methods that evidence starts at low (observational) or high (randomized) certainty and is downgraded or upgraded based on predefined domains. The Results reflect these assessments.*

7. Line 194: There is a * and a ** shown in the diagram. Do those mean anything? Should put what they mean in a legend of in the figure description.

   ***Response**: Thank you for pointing this out. We have removed the erroneous symbols and reuploaded the corrected figure.*

8. Line 241, Line 293, and Figure 5: Where is the p=0.044 reported? I do not see it in the figure and why are there two p values being reported for this figure? And while that p value is significant, the other (p=0.49, the one in the figure) is not significant. What is the relevance of this p value versus the other one? Seems to me that this test should be non-significant?

   ***Response**: We thank the reviewer for the comment. The $p = 0.044$ corresponds to the significance of the mean difference in HbA1c between vitamin D–deficient and non-deficient individuals and was not included in the figure, which focuses on effect estimates. The other $p = 0.49$ refers to the I^2 test for heterogeneity. Thus, the two p-values represent different statistical tests and serve distinct purposes.*

9. Line 251 and Figure 6: The p value is said to be 0.53 in the text, but the figure shows that it is 0.0013. Is there a reason for this discrepancy? Should the text state that this is actually a significant finding?

   ***Response**: Thank you. The $p = 0.53$ reported in the text corresponds to the statistical significance of the pooled odds ratio and was not included in the figure. The $p = 0.0013$ shown in Figure 6 reflects the I^2 test for heterogeneity. The association itself remains non-significant, as correctly stated in the text.*

10. Line 267: You've been reporting CI, I, and p values so far but here you introduce t^2 in place of the CI, is there a reason that this value is now suddenly relevant? Should you be reporting this value for the other tests as well?

    ***Response**: Thank you. The τ^2 value reported here represents the estimated between-study variance in the mixed-effects meta-regression model, which is standard practice for quantifying residual heterogeneity in this context. It differs from a confidence interval and*

is specific to meta-regression analyses. For consistency, other pooled analyses report I^2 and p-values, which are more commonly used for standard meta-analyses.

11. Line 268: Where did this QM value come from? I'm not sure what that is reporting or why it's relevant over what you have been reporting so far.

    ***Response**: Thank you. The QM value represents the omnibus test for moderators in the mixed-effects meta-regression, assessing whether the included study-level characteristics (publication year and sample size) collectively explain heterogeneity in VDD prevalence. It is specific to meta-regression and complements the τ^2, I^2, and p-values reported in standard meta-analyses.*

12. Line 293: Cite figure 5

    ***Response**: We thank the reviewer for the suggestion. The figure has already been cited in the Results section, which is the standard practice for reporting figure references. To maintain consistency and avoid duplication, we have retained the figure citation in the Results only.*

13. General figure comment: Is there a reason tests for figures 2, 3, 4 on use a random effect model and figure 5 and 6 tests have both a random effect and common effect model?

    ***Response**: We thank the reviewer for the comment. Figures 2, 3, and 4 report pooled prevalence estimates, which are typically analyzed using a random-effects model due to the substantial heterogeneity across studies. Figures 5 and 6, which assess associations (mean differences and odds ratios), include both random-effects and fixed-effect (common-effect) estimates to provide a comprehensive view of the results, especially given the small number of studies in these analyses. The random-effects model accounts for between-study variability, while the fixed-effect model provides a reference assuming a common effect across studies. This approach is standard when effect estimates may be sensitive to model choice.*
