# Supplementary material for: Hidden deficiency under bright skies: Vitamin D prevalence and genetic associations in African Type 2 diabetes: A systematic review and meta-analysis
Source: PLoS One. 2026 Jul 24;21(7):e0354518. doi: 10.1371/journal.pone.0354518 (PMC13399315; doi:10.1371/journal.pone.0354518)
Supplement: Attachment — Submitted filename: Response_to_Reviewers_auresp_2.pdf [file pone.0354518.s013.pdf]

Dear Editor-in-Chief,

Thank you for coordinating the review of our manuscript. We are grateful that the first round of revisions was favorably received and for the reviewers' continued constructive feedback. We have carefully addressed all remaining concerns and have prepared a point-by-point response, with revisions highlighted in *green* within the manuscript. The manuscript has been updated accordingly to reflect these changes.

Journal Requirements

1. If the reviewer comments include a recommendation to cite specific previously published works, please review and evaluate these publications to determine whether they are relevant and should be cited. There is no requirement to cite these works unless the editor has indicated otherwise.

   ***Response**: Thank you. While no specific references were suggested, we have added relevant citations after careful review to support the updated content.*

2. Please review your reference list to ensure that it is complete and correct. If you have cited papers that have been retracted, please include the rationale for doing so in the manuscript text, or remove these references and replace them with relevant current references. Any changes to the reference list should be mentioned in the rebuttal letter that accompanies your revised manuscript. If you need to cite a retracted article, indicate the article's retracted status in the References list and also include a citation and full reference for the retraction notice.

   ***Response**: Thank you for this comment. We have carefully reviewed the reference list and confirmed that it is complete and accurate. We did not identify any retracted articles among the cited references; therefore, no changes were required.*

Reviewer #1

I have carefully reviewed the revised manuscript and the authors' point-by-point responses to the reviewers' comments. The authors have done an excellent job of addressing all the concerns and suggestions raised during the initial review stage. The newly added subgroup analyses and meta-regressions regarding vitamin D definitions successfully clarify the analytical approach, and the limitations of the high heterogeneity and genetic findings are now balanced and scientifically sound. Additionally, providing the extracted dataset and R scripts as

supplementary material perfectly aligns with PLOS ONE's commitment to data availability and reproducibility. All required modifications have been thoroughly implemented, and the manuscript is now ready for publication. I recommend its acceptance without further revisions.

Response: *Thank you for your careful review, valuable time, and positive feedback on our revised manuscript. We sincerely appreciate your comments and recommendation for acceptance.*

Reviewer #4

The manuscript addresses an important gap in African diabetes research and has improved substantially after revision.

1. The clinical recommendations exceed the strength of evidence. The manuscript states: "Our findings stress the importance of integrating vitamin D assessment into routine diabetes care in Africa". However, this review is based almost entirely on observational studies and does not evaluate intervention effectiveness.

   Response: *Thank you. We have amended the conclusion throughout to align with the observational nature of our evidence and have removed all recommendations for routine screening or supplementation.*

2. The discussion is short and can be improved. The paper repeatedly mentions that Africa has abundant sunlight but high VDD prevalence. This is interesting but not explored deeply. They should discuss skin pigmentation and reduced cutaneous vitamin D synthesis, Urbanization and indoor lifestyles, dietary vitamin D intake

   Response: *Thank you for this important comment. We have now expanded the discussion to more deeply explore the sunlight paradox by incorporating skin pigmentation, urbanization, and dietary factors as key drivers of VDD in African populations.*

3. Clinical significance of HbA1c difference MD = 0.89% HbA1c which is actually clinically meaningful. A discussion on Is a 0.89% HbA1c difference comparable to some pharmacological interventions? and What impact might this have on diabetic complications? should be included to strengthen the discussion section.

   Response: *Thank you for this valuable suggestion. We have expanded the discussion to contextualize the 0.89% HbA1c difference by comparing it to established glucose-lowering agents and quantifying its potential impact on diabetes complications, while maintaining appropriate caution given the observational nature of our evidence.*
