# Supplementary material for: Hidden deficiency under bright skies: Vitamin D prevalence and genetic associations in African Type 2 diabetes: A systematic review and meta-analysis
Source: PLoS One. 2026 Jul 24;21(7):e0354518. doi: 10.1371/journal.pone.0354518 (PMC13399315; doi:10.1371/journal.pone.0354518)
Supplement: Attachment — Submitted filename: Comments_D-25-50640.docx [file pone.0354518.s010.docx]

# My Reports

This manuscript presents a well-designed and timely systematic review and meta-analysis addressing vitamin D deficiency, glycemic control, and VDR polymorphisms among individuals with type 2 diabetes in African populations. The study follows PRISMA 2020 guidelines, has a registered PROSPERO protocol, applies appropriate statistical methods, and addresses an important gap in the literature. Overall, the manuscript is methodologically sound and largely suitable for publication in PLOS ONE, pending minor but targeted revisions aimed at improving clarity and interpretation.

## Comments

1. Definition and standardization of vitamin D deficiency
Vitamin D deficiency is a primary outcome of this review; however, the included studies apply different cut-off values to define deficiency (e.g., <20 ng/mL vs. <30 ng/mL). The manuscript would benefit from a clearer explanation of how this variability was handled analytically. If feasible, a sensitivity or subgroup analysis based on vitamin D cut-off definitions could be considered. Alternatively, this issue should be more explicitly acknowledged as a contributor to between-study heterogeneity in the Limitations section.

2. Interpretation of substantial heterogeneity
The pooled prevalence analysis demonstrates very high heterogeneity (I² >95%). While meta-regression and subgroup analyses were conducted, the Discussion could be strengthened by a more explicit interpretation of the implications of this heterogeneity. In particular, the authors may wish to clarify how such variability affects the generalizability of the findings and their relevance for regional or clinical decision-making in African contexts.

3. Scope and interpretation of genetic findings
The conclusions regarding the lack of association between VDR FokI polymorphism and type 2 diabetes risk are appropriate; however, they would benefit from additional contextualization. Given the small number of genetic studies and the extensive genetic diversity across African populations, the authors are encouraged to explicitly frame these findings as hypothesis-generating and to emphasize the need for larger, population-specific genetic studies.

4. Data availability and reproducibility
The Data Availability Statement is acceptable; however, the authors are encouraged to clarify whether extracted datasets and/or statistical code (e.g., R scripts) could be made available as supplementary material. This would further enhance transparency and reproducibility, in line with PLOS ONE’s open science principles.
