# Supplementary material for: Hidden deficiency under bright skies: Vitamin D prevalence and genetic associations in African Type 2 diabetes: A systematic review and meta-analysis
Source: PLoS One. 2026 Jul 24;21(7):e0354518. doi: 10.1371/journal.pone.0354518 (PMC13399315; doi:10.1371/journal.pone.0354518)
Supplement: Attachment — Submitted filename: Abera et al 2025 reveiw.docx [file pone.0354518.s011.docx]

Pone-D-25-50640

Reviewer Comments

This a review of “Hidden deficiency under bright skies: Vitamin D prevalence and genetic associations in African type 2 Diabetes: A systematic review and meta-analysis.” This study is a review of the current literature that explores the link between the presence of type 2 diabetes mellitus (T2DM) and vitamin D deficiency (VDD) within several African nations. The author’s identified 22 appropriate studies of with 3,447 total individuals from 11 African nations as being relevant to their research question. Their analysis of the relevant papers showed a link between the presence of VDD and T2DM and recommend that reducing VDD could be an effective compliment to diabetes management in Africa. The study is well done and the authors did a thorough job of identifying and screening for relevant studies and in the subsequent analyses of said studies. I have a few general comments and some remarks regarding the writing, all of which are minor.

Line 98: Add spaces between 1 & 2

Line 108-109: The “as well as we have” in this sentence sounds weird consider changing it to “and we have” or something similar

Line 132-133: Is this PRISMA flow diagram the same as what is shown in Figure 1? Could site Figure 1 here

Line 175: change to “explore the source”

Line 177-182: I was not familiar with the GRADE framework that was mentioned in this section and had to look it up. I think this section could use a citation or two about the framework and a little more expansion on how the up/downgrading is established to acquaint the reader with the tool. This extends to the Certainty of Evidence section starting on Line 275. Does the GRADE framework offer a value to place it in the moderate category? Likewise are we assuming that everything starts at the highest category hence your language of “downgraded for substantial heterogeneity”. Based on that it seems that way.

Line 194: There is a * and a ** shown in the diagram. Do those mean anything? Should put what they mean in a legend of in the figure description.

Line 241, Line 293, and Figure 5: Where is the p=0.044 reported? I do not see it in the figure and why are there two p values being reported for this figure? And while that p value is significant, the other (p=0.49, the one in the figure) is not significant. What is the relevance of this p value versus the other one? Seems to me that this test should be non-significant?

Line 251 and Figure 6: The p value is said to be 0.53 in the text, but the figure shows that it is 0.0013. Is there a reason for this discrepancy? Should the text state that this is actually a significant finding?

Line 267: You’ve been reporting CI, I, and p values so far but here you introduce t^2^ in place of the CI, is there a reason that this value is now suddenly relevant? Should you be reporting this value for the other tests as well?

Line 268: Where did this QM value come from? I’m not sure what that is reporting or why it’s relevant over what you have been reporting so far.

Line 293: Cite figure 5

General figure comment: Is there a reason tests for figures 2, 3, 4 on use a random effect model and figure 5 and 6 tests have both a random effect and common effect model?
