# Supplementary material for: Hidden deficiency under bright skies: Vitamin D prevalence and genetic associations in African Type 2 diabetes: A systematic review and meta-analysis
Source: PLoS One. 2026 Jul 24;21(7):e0354518. doi: 10.1371/journal.pone.0354518 (PMC13399315; doi:10.1371/journal.pone.0354518)
Supplement: Peer review history

# Peer review: Decision Letter 0

**DOI:** 10.1371/journal.pone.0354518.r001

**Authors:** Akif Altınbas

16 Mar 2026

PONE-D-25-50640

Hidden Deficiency under Bright Skies: Vitamin D Prevalence and Genetic Associations in African Type 2 Diabetes: A Systematic Review and Meta-Analysis

PLOS One

Dear Dr. Abera,

Thank you for submitting your manuscript to PLOS ONE. After careful consideration, we feel that it has merit but does not fully meet PLOS ONE’s publication criteria as it currently stands. Therefore, we invite you to submit a revised version of the manuscript that addresses the points raised during the review process.

Please submit your revised manuscript by Apr 30 2026 11:59PM. If you will need more time than this to complete your revisions, please reply to this message or contact the journal office at plosone@plos.org. When you're ready to submit your revision, log on to https://www.editorialmanager.com/pone/ and select the 'Submissions Needing Revision' folder to locate your manuscript file.

Please include the following items when submitting your revised manuscript:

- A letter that responds to each point raised by the academic editor and reviewer(s). You should upload this letter as a separate file labeled 'Response to Reviewers'.
- A marked-up copy of your manuscript that highlights changes made to the original version. You should upload this as a separate file labeled 'Revised Manuscript with Track Changes'.
- An unmarked version of your revised paper without tracked changes. You should upload this as a separate file labeled 'Manuscript'.

If you would like to make changes to your financial disclosure, please include your updated statement in your cover letter. Guidelines for resubmitting your figure files are available below the reviewer comments at the end of this letter.

If applicable, we recommend that you deposit your laboratory protocols in protocols.io to enhance the reproducibility of your results. Protocols.io assigns your protocol its own identifier (DOI) so that it can be cited independently in the future. For instructions see: https://journals.plos.org/plosone/s/submission-guidelines#loc-laboratory-protocols. Additionally, PLOS ONE offers an option for publishing peer-reviewed Lab Protocol articles, which describe protocols hosted on protocols.io. Read more information on sharing protocols at https://plos.org/protocols?utm_medium=editorial-email&utm_source=authorletters&utm_campaign=protocols.

We look forward to receiving your revised manuscript.

Kind regards,

Akif Altınbas, M.D.

Academic Editor

PLOS One

Journal Requirements:

When submitting your revision, we need you to address these additional requirements.

1. Please ensure that your manuscript meets PLOS ONE's style requirements, including those for file naming. The PLOS ONE style templates can be found at

https://journals.plos.org/plosone/s/file?id=wjVg/PLOSOne_formatting_sample_main_body.pdf and

https://journals.plos.org/plosone/s/file?id=ba62/PLOSOne_formatting_sample_title_authors_affiliations.pdf

2. Thank you for stating the following in the Competing Interests section:

The authors report no conflicts of interest in this work.

Please confirm that this does not alter your adherence to all PLOS ONE policies on sharing data and materials, by including the following statement: "This does not alter our adherence to PLOS ONE policies on sharing data and materials.” (as detailed online in our guide for authors http://journals.plos.org/plosone/s/competing-interests). If there are restrictions on sharing of data and/or materials, please state these. Please note that we cannot proceed with consideration of your article until this information has been declared.

Please include your updated Competing Interests statement in your cover letter; we will change the online submission form on your behalf.

3. If the reviewer comments include a recommendation to cite specific previously published works, please review and evaluate these publications to determine whether they are relevant and should be cited. There is no requirement to cite these works unless the editor has indicated otherwise.

[Note: HTML markup is below. Please do not edit.]

Reviewers' comments:

Reviewer's Responses to Questions

**Comments to the Author**

1. Is the manuscript technically sound, and do the data support the conclusions?

The manuscript must describe a technically sound piece of scientific research with data that supports the conclusions. Experiments must have been conducted rigorously, with appropriate controls, replication, and sample sizes. The conclusions must be drawn appropriately based on the data presented.

Reviewer #1: Yes

Reviewer #2: Yes

Reviewer #3: Yes

**********

2. Has the statistical analysis been performed appropriately and rigorously?

Reviewer #1: Yes

Reviewer #2: No

Reviewer #3: Yes

**********

3. Have the authors made all data underlying the findings in their manuscript fully available?

The PLOS Data policy requires authors to make all data underlying the findings described in their manuscript fully available without restriction, with rare exception (please refer to the Data Availability Statement in the manuscript PDF file). The data should be provided as part of the manuscript or its supporting information, or deposited to a public repository. For example, in addition to summary statistics, the data points behind means, medians and variance measures should be available. If there are restrictions on publicly sharing data—e.g. participant privacy or use of data from a third party—those must be specified.

Reviewer #1: Yes

Reviewer #2: Yes

Reviewer #3: Yes

**********

4. Is the manuscript presented in an intelligible fashion and written in standard English?

PLOS ONE does not copyedit accepted manuscripts, so the language in submitted articles must be clear, correct, and unambiguous. Any typographical or grammatical errors should be corrected at revision, so please note any specific errors here.

Reviewer #1: Yes

Reviewer #2: Yes

Reviewer #3: Yes

**********

5. Review Comments to the Author

Please use the space provided to explain your answers to the questions above. You may also include additional comments for the author, including concerns about dual publication, research ethics, or publication ethics. (Please upload your review as an attachment if it exceeds 20,000 characters)

Reviewer #1: My Reports

This manuscript presents a well-designed and timely systematic review and meta-analysis addressing vitamin D deficiency, glycemic control, and VDR polymorphisms among individuals with type 2 diabetes in African populations. The study follows PRISMA 2020 guidelines, has a registered PROSPERO protocol, applies appropriate statistical methods, and addresses an important gap in the literature. Overall, the manuscript is methodologically sound and largely suitable for publication in PLOS ONE, pending minor but targeted revisions aimed at improving clarity and interpretation.

Comments

1. Definition and standardization of vitamin D deficiency

Vitamin D deficiency is a primary outcome of this review; however, the included studies apply different cut-off values to define deficiency (e.g., <20 ng/mL vs. <30 ng/mL). The manuscript would benefit from a clearer explanation of how this variability was handled analytically. If feasible, a sensitivity or subgroup analysis based on vitamin D cut-off definitions could be considered. Alternatively, this issue should be more explicitly acknowledged as a contributor to between-study heterogeneity in the Limitations section.

2. Interpretation of substantial heterogeneity

The pooled prevalence analysis demonstrates very high heterogeneity (I² >95%). While meta-regression and subgroup analyses were conducted, the Discussion could be strengthened by a more explicit interpretation of the implications of this heterogeneity. In particular, the authors may wish to clarify how such variability affects the generalizability of the findings and their relevance for regional or clinical decision-making in African contexts.

3. Scope and interpretation of genetic findings

The conclusions regarding the lack of association between VDR FokI polymorphism and type 2 diabetes risk are appropriate; however, they would benefit from additional contextualization. Given the small number of genetic studies and the extensive genetic diversity across African populations, the authors are encouraged to explicitly frame these findings as hypothesis-generating and to emphasize the need for larger, population-specific genetic studies.

4. Data availability and reproducibility

The Data Availability Statement is acceptable; however, the authors are encouraged to clarify whether extracted datasets and/or statistical code (e.g., R scripts) could be made available as supplementary material. This would further enhance transparency and reproducibility, in line with PLOS ONE’s open science principles.

Reviewer #2: This systematic review addresses an important and understudied topic — vitamin D deficiency (VDD) in African populations with type 2 diabetes mellitus (T2DM). The authors followed PRISMA and JBI guidelines, pre-registered the protocol, and performed a range of appropriate analyses. However, several major analytical concerns substantially limit the interpretability of the findings and require revision before acceptance.

1. VDD Definition Not Clearly Specified: The paper never clearly states how VDD was defined across studies or whether a unified threshold was applied for pooling. This is a critical methodological detail that must be reported transparently in the methods section or a supplementary table.

2. Case-Control Studies and Prevalence Estimation: Eleven of the 22 included studies used a case-control design, from which prevalence estimates should not be directly calculated, as prevalence in such designs is a function of the sampling ratio rather than true population prevalence. It is unclear whether VDD prevalence estimates were drawn only from the "case" (T2DM) arm of these studies and, if so, how they were handled differently from cross-sectional estimates. The subgroup analysis by study design is appreciated but does not resolve this concern, which should be explicitly addressed in the methods.

3. Inclusion of a Non-Peer-Reviewed Source: Reference 34 (Hasan, Algeria) appears to be a thesis rather than a peer-reviewed publication. Its inclusion should be explicitly justified, and its methodological quality should be assessed transparently, as theses are not subject to the same review process as journal articles.

4. Extreme Heterogeneity: The pooled prevalence of 51% (95% CI: 36–65%) is accompanied by I² = 95.7%, which is extreme. This is not merely a limitation to acknowledge — it fundamentally undermines the validity of a single pooled estimate. When I² is this high, the confidence interval is almost entirely driven by between-study variance (τ²), and the pooled figure of 51% carries very little real estimative value. While the authors acknowledged heterogeneity, conducted subgroup analyses by region and study design, and ran a meta-regression on publication year and sample size, none of these explained the variability. The authors should have explored the most clinically plausible sources of heterogeneity as moderators in the meta-regression — specifically: VDD threshold definition (e.g., <20 ng/mL vs. <30 ng/mL vs. <50 nmol/L), assay method (immunoassay vs. LC-MS/MS), season of blood draw, and diabetes duration. These were mentioned in the discussion as speculative explanations but were never formally tested, which represents a significant analytical gap. Furthermore, in case there is insufficient data to assess confounders, the authors should explicitly discuss whether pooling these studies is even appropriate at this level of heterogeneity, or whether a narrative synthesis would be more honest for certain outcomes.

5. VDR FokI Polymorphism Analysis: Only 4 studies were pooled for the VDR FokI analysis, yielding I² = 80.8% and a confidence interval so wide, making it analytically not meaningful: OR = 1.51 (95% CI: 0.23–9.73). While the GRADE rating of "very low" certainty is appropriate, the language used in the abstract and conclusion do not adequately reflect this uncertainty. For instance, lines 45–46 conclude that "current evidence does not support a clear influence of VDR FokI polymorphisms on T2DM susceptibility". This statement that is too confident given the extreme imprecision. A more accurate conclusion would be that the available evidence is insufficient to draw any meaningful conclusion on this outcome.

6. HbA1c Association: The finding that VDD is associated with higher HbA1c (MD = 0.89%, 95% CI: 0.06–1.72, I² = 0%) is based on only 3 studies. Although the I² = 0% is reassuring, three studies are far too few to draw reliable conclusions, and this result should be treated as preliminary and hypothesis-generating. The confidence interval nearly crosses zero, indicating meaningful imprecision. Despite the appropriate GRADE rating of "low" certainty, the language in the abstract and conclusions present this finding with more confidence than is warranted (lines 291–292: "significantly associated with poorer glycemic control"; line 317: "The significant association between VDD and poorer glycemic control"). This language should be softened accordingly. Additionally, it is unclear whether the three included studies adjusted for key confounders such as diabetes duration, medication use, BMI, or renal impairment — all of which affect vitamin D levels and glycemic control. Unadjusted mean differences in observational studies are highly susceptible to confounding, and this issue is not discussed.

7. Meta-Regression: The meta-regression included only publication year and sample size as covariates across 20 studies. While borderline acceptable in terms of the study-to-covariate ratio, the two covariates chosen are arguably the least likely to explain heterogeneity in VDD prevalence. The failure to include more clinically meaningful predictors such as VDD threshold used, assay type, or study setting as moderators (if data were available in the extracted tables) is a missed opportunity and substantially weakens this section.

8. Line 311: The claim that African nations "contributed only five studies" to a prior global review, despite 10 eligible studies existing before that date, should be supported with a supplementary comparison table that also addresses differences in inclusion/exclusion criteria between the two reviews.

9. References: Line 409: Reference 1 ("Analytical Fact Sheet…") is incomplete — no author, year, or URL is provided. This must be corrected.

10. Line 120: The exclusion criterion referencing "sample sizes too small to generate meaningful estimates" does not specify the cutoff used. This threshold should be defined.

11. Seasonal variation as a potential confounding factor in vitamin D levels is not formally addressed and should be discussed or tested where data permit.

12. Line 323: A period is missing at the end of the sentence.

Reviewer #3: This a review of “Hidden deficiency under bright skies: Vitamin D prevalence and genetic associations in African type 2 Diabetes: A systematic review and meta-analysis.” This study is a review of the current literature that explores the link between the presence of type 2 diabetes mellitus (T2DM) and vitamin D deficiency (VDD) within several African nations. The author’s identified 22 appropriate studies of with 3,447 total individuals from 11 African nations as being relevant to their research question. Their analysis of the relevant papers showed a link between the presence of VDD and T2DM and recommend that reducing VDD could be an effective compliment to diabetes management in Africa. The study is well done and the authors did a thorough job of identifying and screening for relevant studies and in the subsequent analyses of said studies. I have a few general comments and some remarks regarding the writing, all of which are minor.

**********

6. PLOS authors have the option to publish the peer review history of their article (what does this mean?). If published, this will include your full peer review and any attached files.

If you choose “no”, your identity will remain anonymous but your review may still be made public.

**Do you want your identity to be public for this peer review?**  For information about this choice, including consent withdrawal, please see our Privacy Policy.

Reviewer #1: **Yes:**  Fateme Binayi

Reviewer #2: No

Reviewer #3: **Yes:**  John Belcik

**********

[NOTE: If reviewer comments were submitted as an attachment file, they will be attached to this email and accessible via the submission site. Please log into your account, locate the manuscript record, and check for the action link "View Attachments". If this link does not appear, there are no attachment files.]

To ensure your figures meet our technical requirements, please review our figure guidelines: https://journals.plos.org/plosone/s/figures

You may also use PLOS’s free figure tool, NAAS, to help you prepare publication quality figures: https://journals.plos.org/plosone/s/figures#loc-tools-for-figure-preparation.

NAAS will assess whether your figures meet our technical requirements by comparing each figure against our figure specifications.

Attachment Submitted filename: Comments_D-25-50640.docx

Attachment Submitted filename: Abera et al 2025 reveiw.docx

# Author comment: Author response to Decision Letter 1

**DOI:** 10.1371/journal.pone.0354518.r002

17 Mar 2026

Journal Requirements

1. Please ensure that your manuscript meets PLOS ONE's style requirements, including those for file naming.

Response: Thank you for the comment. We have updated the manuscript accordingly to comply with the journal formatting requirements, including the file naming.

2. Thank you for stating the following in the Competing Interests section:

The authors report no conflicts of interest in this work.

Please confirm that this does not alter your adherence to all PLOS ONE policies on sharing data and materials, by including the following statement: "This does not alter our adherence to PLOS ONE policies on sharing data and materials.”

Please include your updated Competing Interests statement in your cover letter; we will change the online submission form on your behalf.

Response: Thank you for the clarification. We have updated the Competing Interests section accordingly in both the manuscript and the cover letter.

3. If the reviewer comments include a recommendation to cite specific previously published works, please review and evaluate these publications to determine whether they are relevant and should be cited. There is no requirement to cite these works unless the editor has indicated otherwise.

Response: We note the editor’s suggestion regarding the citation of previously published works. In line with Reviewer 3’s feedback (comment 6), we have included citations for the GRADE framework in the Methods section to provide readers with guidance on the tool used to assess certainty of evidence. No other additional citations were added.

Reviewer #1:

1. This manuscript presents a well-designed and timely systematic review and meta-analysis addressing vitamin D deficiency, glycemic control, and VDR polymorphisms among individuals with type 2 diabetes in African populations. The study follows PRISMA 2020 guidelines, has a registered PROSPERO protocol, applies appropriate statistical methods, and addresses an important gap in the literature. Overall, the manuscript is methodologically sound and largely suitable for publication in PLOS ONE, pending minor but targeted revisions aimed at improving clarity and interpretation.

Response: We thank the reviewer for the positive and constructive feedback. We have addressed the suggested revisions to improve clarity and interpretation as indicated.

2. Definition and standardization of vitamin D deficiency

Vitamin D deficiency is a primary outcome of this review; however, the included studies apply different cut-off values to define deficiency (e.g., <20 ng/mL vs. <30 ng/mL). The manuscript would benefit from a clearer explanation of how this variability was handled analytically. If feasible, a sensitivity or subgroup analysis based on vitamin D cut-off definitions could be considered. Alternatively, this issue should be more explicitly acknowledged as a contributor to between-study heterogeneity in the Limitations section.

Response: Thank you for your comment. We have now addressed the variability in vitamin D deficiency cutoffs by adding the distribution of cutoff definitions in the Study characteristics section, conducting sensitivity analyses showing no significant association between cutoffs and study characteristics, and acknowledging this variation as a source of clinical heterogeneity in the Limitations section.

3. Interpretation of substantial heterogeneity

The pooled prevalence analysis demonstrates very high heterogeneity (I² >95%). While meta-regression and subgroup analyses were conducted, the Discussion could be strengthened by a more explicit interpretation of the implications of this heterogeneity. In particular, the authors may wish to clarify how such variability affects the generalizability of the findings and their relevance for regional or clinical decision-making in African contexts.

Response: We thank the reviewer for the comment. We have included a statement in the Discussion section under Limitations addressing the high heterogeneity and its implications for generalizability and clinical relevance in African populations.

4. Scope and interpretation of genetic findings

The conclusions regarding the lack of association between VDR FokI polymorphism and type 2 diabetes risk are appropriate; however, they would benefit from additional contextualization. Given the small number of genetic studies and the extensive genetic diversity across African populations, the authors are encouraged to explicitly frame these findings as hypothesis-generating and to emphasize the need for larger, population-specific genetic studies.

Response: Thank you for the suggestion. We have revised the Conclusion to frame the findings on VDR FokI polymorphism as hypothesis-generating and emphasize the need for larger, population-specific genetic studies across Africa.

5. Data availability and reproducibility

The Data Availability Statement is acceptable; however, the authors are encouraged to clarify whether extracted datasets and/or statistical code (e.g., R scripts) could be made available as supplementary material. This would further enhance transparency and reproducibility, in line with PLOS ONE’s open science principles.

Response: Thank you for the suggestion. We have included a supplementary file containing both the extracted dataset and the complete R script used for all analyses reported in the manuscript.

Reviewer #2:

1. This systematic review addresses an important and understudied topic — vitamin D deficiency (VDD) in African populations with type 2 diabetes mellitus (T2DM). The authors followed PRISMA and JBI guidelines, pre-registered the protocol, and performed a range of appropriate analyses. However, several major analytical concerns substantially limit the interpretability of the findings and require revision before acceptance.

Response: We thank the reviewer for recognizing the importance of this topic and for acknowledging the methodological rigor of our work. We appreciate the constructive feedback and address the analytical concerns raised to improve the clarity and interpretability of our findings.

2. VDD Definition Not Clearly Specified: The paper never clearly states how VDD was defined across studies or whether a unified threshold was applied for pooling. This is a critical methodological detail that must be reported transparently in the methods section or a supplementary table.

Response: Thank you for your comment. We have addressed the VDD definition by: (1) adding the distribution of cutoff definitions in the Study characteristics section, (2) including each study's specific cutoff in Table 1, (3) conducting sensitivity analyses showing no significant association between cutoffs and study characteristics, and (4) acknowledging this variation as a source of clinical heterogeneity in the Limitations section.

3. Case-Control Studies and Prevalence Estimation: Eleven of the 22 included studies used a case-control design, from which prevalence estimates should not be directly calculated, as prevalence in such designs is a function of the sampling ratio rather than true population prevalence. It is unclear whether VDD prevalence estimates were drawn only from the "case" (T2DM) arm of these studies and, if so, how they were handled differently from cross-sectional estimates. The subgroup analysis by study design is appreciated but does not resolve this concern, which should be explicitly addressed in the methods.

Response: We thank the reviewer for this important methodological observation. For case–control studies, prevalence estimates were calculated using only the T2DM (‘case’) group to ensure that the estimates reflect the target population. For example, Abdelsadek 2018 included 60 T2DM patients and 20 controls, with 73.3 % of cases being vitamin D deficient. Similarly, Fondjo 2017 reported 92.4 % deficiency among 118 T2DM cases versus 60.2 % in controls; only the cases were included in our pooled prevalence analysis. The revised Methods section now explicitly describes how prevalence estimates from case–control studies were handled.

4. Inclusion of a Non-Peer-Reviewed Source: Reference 34 (Hasan, Algeria) appears to be a thesis rather than a peer-reviewed publication. Its inclusion should be explicitly justified, and its methodological quality should be assessed transparently, as these are not subject to the same review process as journal articles.

Response: Thank you for this observation. Reference 34 (Hasan, Algeria) is a thesis included to provide geographical representation of VDD prevalence in Algeria. Its methodological quality was assessed using the same Newcastle–Ottawa Scale applied to all included studies, and it received the lowest score (5 stars) compared with the other cross-sectional studies, which is now noted in the Methods section for transparency.

5. Extreme Heterogeneity: The pooled prevalence of 51% (95% CI: 36–65%) is accompanied by I² = 95.7%, which is extreme. This is not merely a limitation to acknowledge — it fundamentally undermines the validity of a single pooled estimate. When I² is this high, the confidence interval is almost entirely driven by between-study variance (τ²), and the pooled figure of 51% carries very little real estimative value. While the authors acknowledged heterogeneity, conducted subgroup analyses by region and study design, and ran a meta-regression on publication year and sample size, none of these explained the variability. The authors should have explored the most clinically plausible sources of heterogeneity as moderators in the meta-regression — specifically: VDD threshold definition (e.g., <20 ng/mL vs. <30 ng/mL vs. <50 nmol/L), assay method (immunoassay vs. LC-MS/MS), season of blood draw, and diabetes duration. These were mentioned in the discussion as speculative explanations but were never formally tested, which represents a significant analytical gap. Furthermore, in case there is insufficient data to assess confounders, the authors should explicitly discuss whether pooling these studies is even appropriate at this level of heterogeneity, or whether a narrative synthesis would be more honest for certain outcomes.

Response: We thank the reviewer for highlighting the issue of extreme heterogeneity. We acknowledge that the pooled prevalence estimate (I² = 95.7%) reflects substantial between-study variability. In addition to the preexisting subgroup analyses, we conducted formal subgroup analyses and meta-regression by vitamin D cutoff definition. While the full subgroup analysis showed significant differences (p = 0.002), a binary comparison of <20 ng/mL versus ≥20 ng/mL revealed no significant difference (p = 0.373), and meta-regression treating the cutoff as a continuous variable confirmed it did not explain heterogeneity (p = 0.473). These analyses are now included in the revised manuscript.

Other clinically plausible moderators suggested by the reviewer (assay method, season of blood draw, and diabetes duration) were insufficiently or inconsistently reported across studies and therefore could not be formally tested. We have clarified this limitation in the manuscript.

We agree that the high heterogeneity limits the interpretability of a single pooled estimate; however, synthesizing the available evidence remains valuable for highlighting the substantial variability in VDD prevalence across African T2DM populations and for identifying important methodological gaps that future studies should address.

6. VDR FokI Polymorphism Analysis: Only 4 studies were pooled for the VDR FokI analysis, yielding I² = 80.8% and a confidence interval so wide, making it analytically not meaningful: OR = 1.51 (95% CI: 0.23–9.73). While the GRADE rating of "very low" certainty is appropriate, the language used in the abstract and conclusion do not adequately reflect this uncertainty. For instance, lines 45–46 conclude that "current evidence does not support a clear influence of VDR FokI polymorphisms on T2DM susceptibility". This statement that is too confident given the extreme imprecision. A more accurate conclusion would be that the available evidence is insufficient to draw any meaningful conclusion on this outcome.

Response: Thank you for this observation. While the original wording suggested a lack of clear evidence, we agree that it may be interpreted as implying absence of effect. We have therefore revised the Abstract and Conclusion to more clearly reflect the uncertainty, stating that the current evidence is insufficient to draw meaningful conclusions regarding the association between VDR FokI polymorphisms and T2DM susceptibility.

7. HbA1c Association: The finding that VDD is associated with higher HbA1c (MD = 0.89%, 95% CI: 0.06–1.72, I² = 0%) is based on only 3 studies. Although the I² = 0% is reassuring, three studies are far too few to draw reliable conclusions, and this result should be treated as preliminary and hypothesis-generating. The confidence interval nearly crosses zero, indicating meaningful imprecision. Despite the appropriate GRADE rating of "low" certainty, the language in the abstract and conclusions present this finding with more confidence than is warranted (lines 291–292: "significantly associated with poorer glycemic control"; line 317: "The significant association between VDD and poorer glycemic control"). This language should be softened accordingly. Additionally, it is unclear whether the three included studies adjusted for key confounders such as diabetes duration, medication use, BMI, or renal impairment — all of which affect vitamin D levels and glycemic control. Unadjusted mean differences in observational studies are highly susceptible to confounding, and this issue is not discussed.

Response: Thank you for this important comment. We have revised the Discussion to better reflect the uncertainty of this finding. Specifically, we clarified that the HbA1c association is based on a small number of studies and should be interpreted with caution. We also acknowledged the lack of consistent adjustment for key confounders (such as diabetes duration, medication use, BMI, and renal function), and emphasized that this result should be considered hypothesis-generating rather than conclusive.

8. Meta-Regression: The meta-regression included only publication year and sample size as covariates across 20 studies. While borderline acceptable in terms of the study-to-covariate ratio, the two covariates chosen are arguably the least likely to explain heterogeneity in VDD prevalence. The failure to include more clinically meaningful predictors such as VDD threshold used, assay type, or study setting as moderators (if data were available in the extracted tables) is a missed opportunity and substantially weakens this section.

Response: We thank the reviewer for this insightful comment. In addition to the original meta-regression on publication year and sample size, we conducted meta-regression using vitamin D cutoff thresholds as a continuous variable. This analysis showed that variation in cutoff values did not significantly explain heterogeneity (p = 0.473). We acknowledge that other clinically relevant moderators, such as assay type or study setting, could not be formally assessed due to limited reporting in the primary studies. We have updated the Methods and Results sections to describe this additional analysis and explicitly note these limitations.

9. Line 311: The claim that African nations "contributed only five studies" to a prior global review, despite 10 eligible studies existing before that date, should be supported with a supplementary comparison table that also addresses differences in inclusion/exclusion criteria between the two reviews.

Response: We thank the reviewer for this suggestion. Instead of creating a separate table, we have included the relevant information on African studies from the prior global review directly in our supplementary extracted studies table (S5 File). This approach allows readers to easily compare study coverage between reviews.

10. References: Line 409: Reference 1 ("Analytical Fact Sheet…") is incomplete — no author, year, or URL is provided. This must be corrected.

Response: We thank the reviewer for noticing this. The reference has been updated accordingly

11. Line 120: The exclusion criterion referencing "sample sizes

Attachment Submitted filename: Response to Reviewers.pdf

# Peer review: Decision Letter 1

**DOI:** 10.1371/journal.pone.0354518.r003

**Authors:** Akif Altınbas

6 Jul 2026

PONE-D-25-50640R1

Hidden deficiency under bright skies: Vitamin D prevalence and genetic associations in African Type 2 diabetes: A systematic review and meta-analysis

PLOS One

Dear Dr. Abera,

Thank you for submitting your manuscript to PLOS ONE. After careful consideration, we feel that it has merit but does not fully meet PLOS ONE’s publication criteria as it currently stands. Therefore, we invite you to submit a revised version of the manuscript that addresses the points raised during the review process.

Please submit your revised manuscript by Aug 20 2026 11:59PM. If you will need more time than this to complete your revisions, please reply to this message or contact the journal office at plosone@plos.org. When you're ready to submit your revision, log on to https://www.editorialmanager.com/pone/ and select the 'Submissions Needing Revision' folder to locate your manuscript file.

Please include the following items when submitting your revised manuscript:

- A letter that responds to each point raised by the academic editor and reviewer(s). You should upload this letter as a separate file labeled 'Response to Reviewers'.
- A marked-up copy of your manuscript that highlights changes made to the original version. You should upload this as a separate file labeled 'Revised Manuscript with Track Changes'.
- An unmarked version of your revised paper without tracked changes. You should upload this as a separate file labeled 'Manuscript'.

If you would like to make changes to your financial disclosure, please include your updated statement in your cover letter. Guidelines for resubmitting your figure files are available below the reviewer comments at the end of this letter.

If applicable, we recommend that you deposit your laboratory protocols in protocols.io to enhance the reproducibility of your results. Protocols.io assigns your protocol its own identifier (DOI) so that it can be cited independently in the future. For instructions see: https://journals.plos.org/plosone/s/submission-guidelines#loc-laboratory-protocols. Additionally, PLOS ONE offers an option for publishing peer-reviewed Lab Protocol articles, which describe protocols hosted on protocols.io. Read more information on sharing protocols at https://plos.org/protocols?utm_medium=editorial-email&utm_source=authorletters&utm_campaign=protocols.

As the corresponding author, your ORCID iD is verified in the submission system and will appear in the published article. PLOS supports the use of ORCID, and we encourage all coauthors to register for an ORCID iD and use it as well. Please encourage your coauthors to verify their ORCID iD within the submission system before final acceptance, as unverified ORCID iDs will not appear in the published article. *Only*  the individual author can complete the verification step; PLOS staff *cannot*  verify ORCID iDs on behalf of authors.

We look forward to receiving your revised manuscript.

Kind regards,

Akif Altınbas, M.D.

Academic Editor

PLOS One

**Journal Requirements:**

1. If the reviewer comments include a recommendation to cite specific previously published works, please review and evaluate these publications to determine whether they are relevant and should be cited. There is no requirement to cite these works unless the editor has indicated otherwise.

2. Please review your reference list to ensure that it is complete and correct. If you have cited papers that have been retracted, please include the rationale for doing so in the manuscript text, or remove these references and replace them with relevant current references. Any changes to the reference list should be mentioned in the rebuttal letter that accompanies your revised manuscript. If you need to cite a retracted article, indicate the article’s retracted status in the References list and also include a citation and full reference for the retraction notice.

[Note: HTML markup is below. Please do not edit.]

Reviewers' comments:

Reviewer's Responses to Questions

**Comments to the Author**

1. If the authors have adequately addressed your comments raised in a previous round of review and you feel that this manuscript is now acceptable for publication, you may indicate that here to bypass the “Comments to the Author” section, enter your conflict of interest statement in the “Confidential to Editor” section, and submit your "Accept" recommendation.

Reviewer #1: All comments have been addressed

Reviewer #4: (No Response)

**********

2. Is the manuscript technically sound, and do the data support the conclusions?

The manuscript must describe a technically sound piece of scientific research with data that supports the conclusions. Experiments must have been conducted rigorously, with appropriate controls, replication, and sample sizes. The conclusions must be drawn appropriately based on the data presented.

Reviewer #1: Yes

Reviewer #4: Yes

**********

3. Has the statistical analysis been performed appropriately and rigorously?

Reviewer #1: Yes

Reviewer #4: Yes

**********

4. Have the authors made all data underlying the findings in their manuscript fully available?

The PLOS Data policy requires authors to make all data underlying the findings described in their manuscript fully available without restriction, with rare exception (please refer to the Data Availability Statement in the manuscript PDF file). The data should be provided as part of the manuscript or its supporting information, or deposited to a public repository. For example, in addition to summary statistics, the data points behind means, medians and variance measures should be available. If there are restrictions on publicly sharing data—e.g. participant privacy or use of data from a third party—those must be specified.

Reviewer #1: Yes

Reviewer #4: Yes

**********

5. Is the manuscript presented in an intelligible fashion and written in standard English?

PLOS ONE does not copyedit accepted manuscripts, so the language in submitted articles must be clear, correct, and unambiguous. Any typographical or grammatical errors should be corrected at revision, so please note any specific errors here.

Reviewer #1: Yes

Reviewer #4: Yes

**********

6. Review Comments to the Author

Please use the space provided to explain your answers to the questions above. You may also include additional comments for the author, including concerns about dual publication, research ethics, or publication ethics. (Please upload your review as an attachment if it exceeds 20,000 characters)

Reviewer #1: I have carefully reviewed the revised manuscript and the authors' point-by-point responses to the reviewers' comments. The authors have done an excellent job of addressing all the concerns and suggestions raised during the initial review stage. The newly added subgroup analyses and meta-regressions regarding vitamin D definitions successfully clarify the analytical approach, and the limitations of the high heterogeneity and genetic findings are now balanced and scientifically sound. Additionally, providing the extracted dataset and R scripts as supplementary material perfectly aligns with PLOS ONE's commitment to data availability and reproducibility. All required modifications have been thoroughly implemented, and the manuscript is now ready for publication. I recommend its acceptance without further revisions.

Reviewer #4: The manuscript addresses an important gap in African diabetes research and has improved substantially after revision.

1- The clinical recommendations exceed the strength of evidence. The manuscript states: "Our findings stress the importance of integrating vitamin D assessment into routine diabetes care in Africa" . However, this review is based almost entirely on observational studies and does not evaluate intervention effectiveness.

2- The discussion is short and can be improved. The paper repeatedly mentions that Africa has abundant sunlight but high VDD prevalence. This is interesting but not explored deeply. They should discuss skin pigmentation and reduced cutaneous vitamin D synthesis, Urbanization and indoor lifestyles, dietary vitamin D intake

3- Clinical significance of HbA1c difference MD = 0.89% HbA1c which is actually clinically meaningful. A discussion on Is a 0.89% HbA1c difference comparable to some pharmacological interventions? and What impact might this have on diabetic complications? should be included to strengthen the discussion section

**********

7. PLOS authors have the option to publish the peer review history of their article (what does this mean?). If published, this will include your full peer review and any attached files.

If you choose “no”, your identity will remain anonymous but your review may still be made public.

**Do you want your identity to be public for this peer review?**  For information about this choice, including consent withdrawal, please see our Privacy Policy.

Reviewer #1: **Yes:**  fateme binayi

Reviewer #4: **Yes:**  Amira Raudhah Abdullah

**********

[NOTE: If reviewer comments were submitted as an attachment file, they will be attached to this email and accessible via the submission site. Please log into your account, locate the manuscript record, and check for the action link "View Attachments". If this link does not appear, there are no attachment files.]

To ensure your figures meet our technical requirements, please review our figure guidelines: https://journals.plos.org/plosone/s/figures

You may also use PLOS’s free figure tool, NAAS, to help you prepare publication quality figures: https://journals.plos.org/plosone/s/figures#loc-tools-for-figure-preparation.

NAAS will assess whether your figures meet our technical requirements by comparing each figure against our figure specifications.

# Author comment: Author response to Decision Letter 2

**DOI:** 10.1371/journal.pone.0354518.r004

8 Jul 2026

Journal Requirements

1. If the reviewer comments include a recommendation to cite specific previously published works, please review and evaluate these publications to determine whether they are relevant and should be cited. There is no requirement to cite these works unless the editor has indicated otherwise.

Response: Thank you. While no specific references were suggested, we have added relevant citations after careful review to support the updated content.

2. Please review your reference list to ensure that it is complete and correct. If you have cited papers that have been retracted, please include the rationale for doing so in the manuscript text, or remove these references and replace them with relevant current references. Any changes to the reference list should be mentioned in the rebuttal letter that accompanies your revised manuscript. If you need to cite a retracted article, indicate the article’s retracted status in the References list and also include a citation and full reference for the retraction notice.

Response: Thank you for this comment. We have carefully reviewed the reference list and confirmed that it is complete and accurate. We did not identify any retracted articles among the cited references; therefore, no changes were required.

Reviewer #1

I have carefully reviewed the revised manuscript and the authors' point-by-point responses to the reviewers' comments. The authors have done an excellent job of addressing all the concerns and suggestions raised during the initial review stage. The newly added subgroup analyses and meta-regressions regarding vitamin D definitions successfully clarify the analytical approach, and the limitations of the high heterogeneity and genetic findings are now balanced and scientifically sound. Additionally, providing the extracted dataset and R scripts as supplementary material perfectly aligns with PLOS ONE's commitment to data availability and reproducibility. All required modifications have been thoroughly implemented, and the manuscript is now ready for publication. I recommend its acceptance without further revisions.

Response: Thank you for your careful review, valuable time, and positive feedback on our revised manuscript. We sincerely appreciate your comments and recommendation for acceptance.

Reviewer #4

The manuscript addresses an important gap in African diabetes research and has improved substantially after revision.

1. The clinical recommendations exceed the strength of evidence. The manuscript states: "Our findings stress the importance of integrating vitamin D assessment into routine diabetes care in Africa”. However, this review is based almost entirely on observational studies and does not evaluate intervention effectiveness.

Response: Thank you. We have amended the conclusion throughout to align with the observational nature of our evidence and have removed all recommendations for routine screening or supplementation.

2. The discussion is short and can be improved. The paper repeatedly mentions that Africa has abundant sunlight but high VDD prevalence. This is interesting but not explored deeply. They should discuss skin pigmentation and reduced cutaneous vitamin D synthesis, Urbanization and indoor lifestyles, dietary vitamin D intake

Response: Thank you for this important comment. We have now expanded the discussion to more deeply explore the sunlight paradox by incorporating skin pigmentation, urbanization, and dietary factors as key drivers of VDD in African populations.

3. Clinical significance of HbA1c difference MD = 0.89% HbA1c which is actually clinically meaningful. A discussion on Is a 0.89% HbA1c difference comparable to some pharmacological interventions? and What impact might this have on diabetic complications? should be included to strengthen the discussion section.

Response: Thank you for this valuable suggestion. We have expanded the discussion to contextualize the 0.89% HbA1c difference by comparing it to established glucose-lowering agents and quantifying its potential impact on diabetes complications, while maintaining appropriate caution given the observational nature of our evidence.

Attachment Submitted filename: Response_to_Reviewers_auresp_2.pdf

# Editor report: Decision Letter 2

**DOI:** 10.1371/journal.pone.0354518.r005

**Authors:** Akif Altınbas

9 Jul 2026

Hidden deficiency under bright skies: Vitamin D prevalence and genetic associations in African Type 2 diabetes: A systematic review and meta-analysis

PONE-D-25-50640R2

Dear Dr. Abera,

We’re pleased to inform you that your manuscript has been judged scientifically suitable for publication and will be formally accepted for publication once it meets all outstanding technical requirements.

Within one week, you’ll receive an e-mail detailing the required amendments. When these have been addressed, you’ll receive a formal acceptance letter and your manuscript will be scheduled for publication.

An invoice will be generated when your article is formally accepted. Please note, if your institution has a publishing partnership with PLOS and your article meets the relevant criteria, all or part of your publication costs will be covered. Please make sure your user information is up-to-date by logging into Editorial Manager at Editorial Manager® and clicking the ‘Update My Information' link at the top of the page. For questions related to billing, please contact billing support.

If your institution or institutions have a press office, please notify them about your upcoming paper to help maximize its impact. If they’ll be preparing press materials, please inform our press team as soon as possible -- no later than 48 hours after receiving the formal acceptance. Your manuscript will remain under strict press embargo until 2 pm Eastern Time on the date of publication. For more information, please contact onepress@plos.org.

Kind regards,

Akif Altınbas, M.D.

Academic Editor

PLOS One

Additional Editor Comments (optional):

Reviewers' comments:

# Editor report: Acceptance letter

**DOI:** 10.1371/journal.pone.0354518.r006

**Authors:** Akif Altınbas

PONE-D-25-50640R2

PLOS One

Dear Dr. Abera,

I'm pleased to inform you that your manuscript has been deemed suitable for publication in PLOS One. Congratulations! Your manuscript is now being handed over to our production team.

At this stage, our production department will prepare your paper for publication. This includes ensuring the following:

* All references, tables, and figures are properly cited

* All relevant supporting information is included in the manuscript submission,

* There are no issues that prevent the paper from being properly typeset

You will receive further instructions from the production team, including instructions on how to review your proof when it is ready. Please keep in mind that we are working through a large volume of accepted articles, so please give us a few days to review your paper and let you know the next and final steps.

Lastly, if your institution or institutions have a press office, please let them know about your upcoming paper now to help maximize its impact. If they'll be preparing press materials, please inform our press team within the next 48 hours. Your manuscript will remain under strict press embargo until 2 pm Eastern Time on the date of publication. For more information, please contact onepress@plos.org.

You will receive an invoice from PLOS for your publication fee after your manuscript has reached the completed accept phase. If you receive an email requesting payment before acceptance or for any other service, this may be a phishing scheme. Learn how to identify phishing emails and protect your accounts at https://explore.plos.org/phishing.

If we can help with anything else, please email us at customercare@plos.org.

Thank you for submitting your work to PLOS ONE and supporting open access.

Kind regards,

PLOS ONE Editorial Office Staff

on behalf of

Assistant Professor Akif Altınbas

Academic Editor

PLOS One
